# Evaluating elimination thresholds and stopping criteria for interventions against the vector-borne macroparasitic disease, lymphatic filariasis, using mathematical modelling

**DOI:** 10.1038/s42003-022-04391-9

**Published:** 2023-02-27

**Authors:** Swarnali Sharma, Morgan E. Smith, Shakir Bilal, Edwin Michael

**Affiliations:** 1 Christian Medical College, IDA Scudder Road, Vellore, Tamil Nadu 632004 India; 2 Department of Biological Sciences, University of Notre Dame, Notre Dame, South Bend, IN USA; 3 Center for Global Health Infectious Disease Research, University of South Florida, Tampa, FL USA

**Keywords:** Statistical methods, Disease model

## Abstract

We leveraged the ability of EPIFIL transmission models fit to field data to evaluate the use of the WHO Transmission Assessment Survey (TAS) for supporting Lymphatic Filariasis (LF) intervention stopping decisions. Our results indicate that understanding the underlying parasite extinction dynamics, particularly the protracted transient dynamics involved in shifts to the extinct state, is crucial for understanding the impacts of using TAS for determining the achievement of LF elimination. These findings warn that employing stopping criteria set for operational purposes, as employed in the TAS strategy, without a full consideration of the dynamics of extinction could seriously undermine the goal of achieving global LF elimination.

## Abstract (web summary)

Mathematical models fit to field data show that protracted transient dynamics may govern the extinction of lymphatic filariasis following interventions and question existing WHO guidelines for assessing elimination of the disease.

## Introduction

Lymphatic filariasis (LF), a highly debilitating mosquito-borne macroparasitic disease of humans, is only one of eight neglected tropical diseases (NTDs) selected by the World Health Organization (WHO) in 1997 for either elimination or eradication as a global public health problem^1^. This resulted in the development of the Global Program to Eliminate Lymphatic Filariasis (GPELF) in 2000, which espoused meeting the goal of eliminating this disease in all 73 endemic countries by 2020 through the application of annual mass drug administration (MDA) maintained over at least 4–6 years^1^. GPELF is not only one of the largest global intervention programs devised to reduce the burden of tropical diseases, but since its origin the program has also made remarkably effective strides in reducing infection prevalences in all the endemic countries in which large-scale nation-wide LF MDA programs have been implemented^2^. This striking success means that many LF programs are now beginning to enter into the endgame or pre-elimination phase of the intervention, with some also considering scaling down MDA to move into a period of post-MDA surveillance that will culminate with a request to WHO to verify the achievement of country-wide elimination of the disease^3^.

While the above progress has highlighted the effectiveness of GPELF for successfully reducing the burden of LF in endemic populations, it has also raised increasing attention on how best to define and implement robust strategies for supporting decisions to end interventions. In 2011, the WHO recommended the use of an epidemiological assessment strategy that comprises conducting a series of infection surveys of entire communities initially, and subsequently of children aged 6–7 years in treated communities, to provide evidence for deciding if parasite transmission has been interrupted and thereby facilitate the making of defensible intervention-stopping decisions^4^. The first survey in this strategy, termed a pre-Transmission Assessment Survey (TAS) or pre-TAS, is to be undertaken in all sentinel and spot check sites of implementation units (IUs)—normally these are districts—which have had at least five effective (>65% coverage) rounds of MDA to evaluate if community-level prevalences of either microfilaraemia (mf) or circulating antigen (CFA) have reached below 1% or 2%, respectively^4^. If either of these targets is met in all the surveyed communities, then an IU is deemed eligible for initiating TAS surveys of children aged 6–7 years sampled from across populations in an IU^5^. In this scheme, the first TAS is designed to be conducted at least 6 months after the final round of MDA to decide if drug treatments can be stopped, while subsequent TAS surveys are conducted (at 2–3 year intervals) to establish the absence of ongoing transmission. Transmission is considered not sustainable when over the period of TAS survey bouts (i.e., at least over a period of 5 years after stopping MDA) the mean antigen (CFA) prevalence in the sampled children drops and remains essentially below half the community-wide CFA threshold mentioned above, i.e., 1% (or equivalently <0.5% mf) in areas where *Anopheles, Culex*, and *Mansonia* species are the main mosquito vectors^4^.

The above strategy has been integrated into national LF programs since 2011, and is widely thought among managers to represent a standardized, statistically rigorous, and easily implementable tool for guiding LF intervention-stopping decisions. However, increasing evidence from the field^6–8^ and results from recent modeling work^9–12^ have called to question whether meeting the stopping criteria (crossing below the 1% mf or 2% CFA threshold) as recommended by WHO would indeed signify the breakage of LF transmission in endemic communities (as indicated by CFA or mf prevalence remaining below 1% or 0.5% respectively in the sampled child subpopulation) and hence if MDA can be safely terminated following this strategy. Specifically, while the available field studies that have evaluated the results of following the stopping protocol above have shown that it may not only not allow reliable assessments of breakage in transmission nor provide a good indication of prevalence levels in older age groups^7,13^, the globally applicable thresholds used in this protocol may also not reflect the likely much lower transmission interruption values and the site-to-site variation estimated for these LF thresholds by data-driven mathematical models^9,10^. Furthermore, the TAS strategy appears to be devised such that the threshold values chosen are sufficiently low to presume that breaching them would lead to transmission interruption, whilst also serving simultaneously to allow the setting and use of sample sizes that are thought to be achievable in the field^14^.

The above suggests that the WHO-recommended LF elimination thresholds and the TAS protocol may be seen to serve as a practical framework for guiding intervention-stopping decisions; however, it is clear given the growing discrepant empirical evidence regarding the impact of using the adopted thresholds that it is also important to determine whether they provide the right results for the right reasons, viz. that meeting the currently set stopping criteria based on the recommended thresholds will indeed lead to cessation of parasite transmission. This concern at its core questions whether crossing the present WHO-set TAS thresholds actually denote the achievement of sustained interruption of LF transmission (defined as noted above specifically as infection found to be consistently below 0.5% mf or 1% CFA in children aged 6–7 years during the TAS phase^3^). If they do not, then as mathematical models and growing evidence from the field show^6–12^, recrudescence of infection can be expected which will seriously thwart the goal of successfully achieving the global elimination of this parasitic disease.

Parasite transmission models aim to encapsulate our understanding of the non-linear, non-additive behavior of a parasitic system, and thus provide powerful tools not only for quantifying the dynamical impact of interventions, but also for investigating the quantitative and qualitative nature of thresholds that signify that transmission in nature has been broken^3,9,10^. Given these, they thus can also serve as a tool for determining if we are getting the right results for the right reasons when addressing a policy objective. In addition, developing and applying data-driven prediction frameworks that allow coupling of information on local system dynamics as provided by field observations with models means that that we would also be able to get scientifically consistent answers for meeting policy objectives reliably well everywhere^9,10^.

In this study, we leverage the ability of mathematical models fit to field data for allowing direct calculations of probabilities of transmission elimination once infection levels are forecast to fall below given thresholds to evaluate: (1) the implications of using the WHO TAS criteria for assessing the achievement of LF transmission interruption, and conversely, (2) the likely probability of transmission recrudescence if elimination of transmission is not achieved. The investigation was carried out by using the deterministic population-based LF model, EPIFIL^9,10^, calibrated to longitudinal human infection data from six field studies that encompass endemic settings typifying the full range of low, moderate, and high LF transmission intensities and which also provided MDA intervention details. Predictions of the impacts of MDA-based interventions are compared by using the WHO-set global TAS versus model-estimated thresholds in these sites in order to highlight the significance of using threshold values more explicitly linked to the underlying localized parasite transmission, and crucially, extinction dynamics, in a setting compared to those more generally or globally set. Finally, we examine the relative effectiveness of currently existing or proposed MDA-based strategies for best ensuring the achievement of LF transmission interruption whilst also suppressing the probability of recrudescence, paying particular attention to the role that supplementary vector control can play for meeting both these outcomes. We further show how these comparisons are useful to identifying the right intervention strategy that could allow the use of the WHO thresholds in case the model-estimated infection thresholds are found to be too difficult to measure readily in the field^9,10^. A major finding arising from this work thus far not fully appreciated in parasite control relates to the role that transient system dynamics near elimination thresholds may have for influencing the length of time to LF transmission extinction. This work demonstrates, for the first time, that understanding the driving processes and impact of this protracted transient state will be key to uncovering the best management options for bringing about effective LF transmission elimination.

## Results

### Estimating local LF models and assessment of predictive performance

**Fig. 1:**
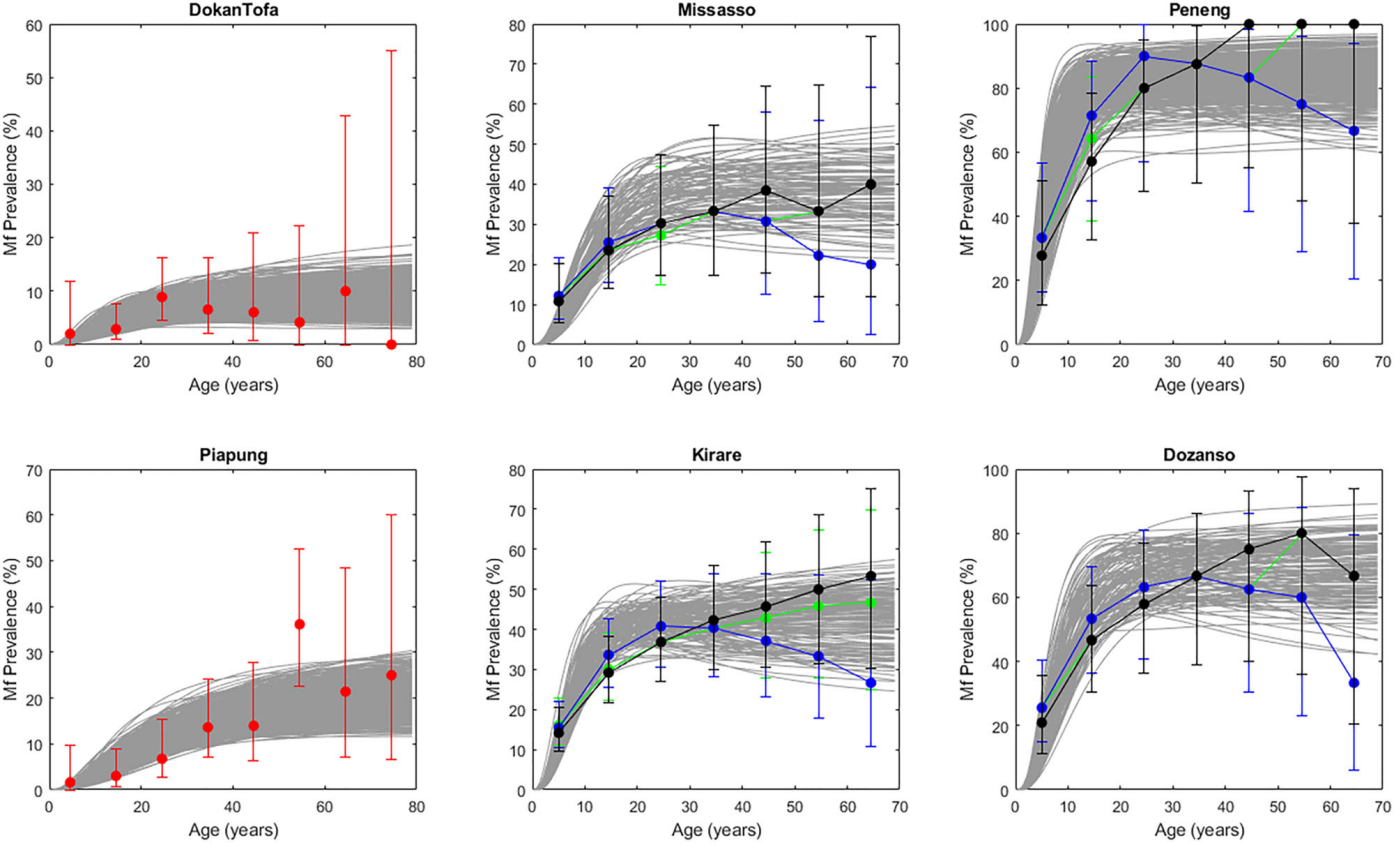
The SIR BM-based model fits to mf prevalence baseline data for two low-prevalence sites (DokanTofa and Piapung), two medium-prevalence sites (Missasso and Kirare), and two high-prevalence sites (Peneng and Dozanso). Red circles indicate the observed age-prevalence data in the case of Dokan Tofa and Piapung, whereas age-stratified prevalence data derived from observed overall community baseline prevalences are shown for linear, plateau and convex age patterns of LF infection by the black, green, and blue curves, respectively, in Missasso, Kirare, Peneng, and Dozanso. Gray lines depict the model predictions for each site. The age-stratified and overall Monte Carlo p-values for the fits to baseline infection data for each of the six study sites were evaluated and are provided in Supplementary Table 3 in Supplementary Information. These show that apart from Piapung and Kirare, good fits were obtained for all other villages. Error bars indicate the 95% confidence intervals for each data point.

We began by assessing the ability of our Bayesian Melding (BM)-based data-model fusion modeling approach for successfully estimating best-fitting LF models based on baseline mf prevalence and the corresponding annual vector transmission intensities observed in each of this study sites (Supplementary Table 2). Note that age-stratified mf prevalences had to be constructed for four of these sites that provided only overall community mf prevalence observations^15,16^, and all mf prevalence values were corrected to reflect sampling of 1 ml blood volumes for carrying out this exercise^15,17^. The curves in Fig. 1 show that the LF models identified for each site based on the Sampling Importance Resampling (SIR) algorithm are able to satisfactorily reproduce the site-varying baseline mf infection prevalence observed for either of these types of data—i.e., whether actually measured (red circles) or constructed (blue/green/black circles joined by lines) (Monte Carlo P values >0.05 for each site except in the case of Piapung and Kirare (Supplementary Table 3)).

**Fig. 2:**
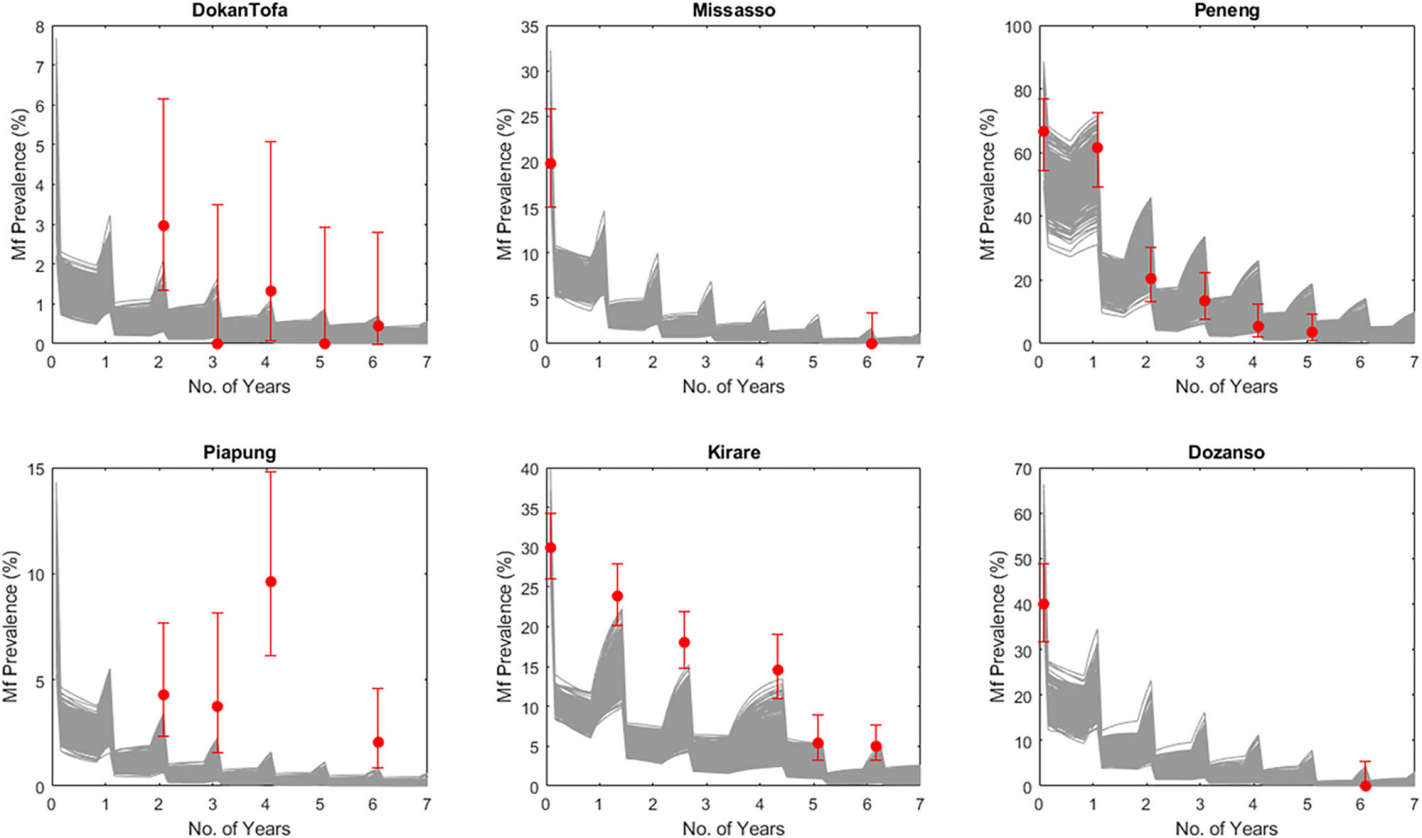
Forecasting impacts of the MDAs carried out in each site on changes in mf prevalence for two low-prevalence sites (DokanTofa and Piapung), two medium-prevalence sites (Missasso and Kirare), and two high-prevalence sites (Peneng and Dozanso). Red circles indicate the observed data and gray lines indicate the model predictions. The overall Monte Carlo p-values for the fits of post-MDA data for the six study sites were evaluated and indicated good fits in all sites (provided in Supplementary Table 3 in Supplementary Information). Error bars indicate the 95% confidence intervals of the data points.

The predictive performances of these locally data-informed models for simulating intervention outcomes were assessed by comparing forecasts of the impacts of the specific MDAs carried out in each site (Supplementary Table 2) for reducing mf prevalence against the corresponding actually observed reductions in prevalences per site (Fig. 2). These comparisons indicate that, as with predictions of site-specific baseline infection prevalences (Fig. 1), and apart from a tendency to overestimate prevalence reductions at some time points in the Piapung and Kirare sites, the estimated locally applicable LF models are also able to mimic the observed changes in the data due to the applied interventions sufficiently well (Monte Carlo *P* values for assessing goodness-of-fit insignificant in each setting >0.05 (Supplementary Table 3)). This ability of the site-specific models to match MDA-driven drops in infection in each setting reasonably well means that parameters other than those related to MDA effects on worm/mf kill and reproductive rates continue to remain stable over at least the MDA periods modeled here, supporting our previous findings regarding the long-term stability of the parameters governing LF transmission in a locality^9,10^. Such transmission robustness engendered through this parameter stability means that system properties, including extinction thresholds estimated using models fit to baseline infection data, are also likely to be stable at least over the intervention periods studied here.

### Estimating timelines and probabilities of elimination due to the annual MDA interventions applied in each site

The numerical forecasts of the timelines made by each site-specific ensemble of 500 best-fiting models (see details of ensemble model construction in “Methods”) for reaching the 1% mf prevalence threshold due to the annual MDAs applied (at the average coverages reported in Supplementary Table 2) in each of the present study sites are given in Supplementary Table 4. These highlight that apart for the case of Piapung the present models are able to reliably predict the required MDA intervention durations for breaching the 1% mf target in each site. Thus, where the number of years predicted by the locality-specific models for achieving the 1% mf threshold were either lower or equal to the actually applied annual MDA rounds, the final prevalences observed (following the applied MDAs) in these sites were found to be lower than the 1% mf prevalence threshold (Supplementary Table 4). This is in contrast to sites (e.g., Kirare, Peneng) in which the model predicted annual MDA durations for reaching the 1% mf threshold are estimated to be longer than the actually applied MDA durations; in this case, the end mf prevalences reached are, as expected, found to be higher than the 1% mf prevalence target (Supplementary Table 4). This matching of predictions with observations futher confirm the conclusion made above that the present locally calibrated models are empirically adequate for capturing the dynamics of the applied MDA control in each site, and thus can serve as reliable tools for addressing the chief objective of this study, viz. evaluating the consequences of applying the WHO TAS thresholds for guiding decision making regarding the site-specific interruption of LF transmission.

Supplementary Table 4 also provides the elimination probabilities over the 5-year TAS period estimated by the localized predictive models following the reduction of mf prevalences below the 1% mf threshold, while Supplementary Table 5 gives these probabilities for the significantly much lower model-estimated 95% elimination probability (EP) mf prevalence thresholds (see detailed definition and derivation of these thresholds and how elimination probabilities are calculated in the “Methods” section) estimated at the annual biting rate (ABR) or at the threshold biting rate (TBR) in each of the present sites. Note as we indicated previously^9^, the mf prevalence 95% EP thresholds estimated at the undisturbed ABR values in each site should be used as targets for quantifying transmission interruption when MDA alone is used, whereas the approximately onefold higher valued thresholds estimated at TBRs should be used as targets for quantifying LF elimination when vector control (VC) is added to MDA. The calculated probabilities show that while achieving the 1% mf prevalence target in each site will require significantly fewer years of annual MDAs (from 3 to 9 years depending on initial endemicity), such achievement will, however, result in either low or moderate chance (1–41%) of transmission interruption associated with a corresponding significant probability of recrudescence (between 15 and 55%) 5 years after crossing this threshold (Supplementary Table 4). By contrast, even though it will take considerably longer (11–19 years) to achieve the much lower 95% EP thresholds (ranging from 0.001 to 0.007%) applicable in each site, the results show that once these thresholds are crossed using the applied annual MDAs, the probability of achieving elimination of transmission will be very high (between 54 and 98%) in each site, except in the case of Piapung village (31%), with the corresponding recrudescence probabilities effectively close to zero in each site (Supplementary Table 5).

### Assessments of the impacts of MDA intervention scenarios with and without the inclusion of vector control on elimination and recrudescence probabilities

We next examined the relative effectiveness of three MDA scenarios with and without inclusion of Vector Control (VC) for accomplishing sustainable LF elimination and reducing likely recrudescence in the present sites. The three MDA-based scenarios chosen for study were: (i) annual MDA, (ii) biannual MDA, and (iii) annual IDA, firstly, with and without inclusion of supplementary VC from the beginning of MDA to when the WHO proposed 1% mf prevalence or model-estimated 95% EP thresholds are breached, and, secondly, after the achievement of these thresholds using MDA continuing with VC alone for either over the 5-year TAS period or for up to 20 years post-stoppage of MDA. The drug combinations applied in each site (i.e., ivermectin and albendazole (IVM+ALB) or diethylcarbamazine and ivermectin (DEC+IVM) (Supplementary Table 2)) were used for simulating the impacts of annual and biannual MDA, respectively, in each site, while the combined effects of IVM, DEC, and ALB were modeled for forecasting the impact of annual IDA. The average MDA coverage and baseline mf prevalence pertaining to each site (as listed in Supplementary Table 2) were used to run these scenario simulations (see “Methods” for simulation details).

The results from carrying out the various intervention simulations are shown in the case of Piapung, Missaso, and Dozanso villages, representing the low, medium and high baseline endemic sites investigated here respectively, for both the 1% (Table 1) and their corresponding site-specific 95% EP mf prevalence thresholds (Table 2). The time in years to cross each respective threshold and the probabilities of achieving elimination or recrudescence following reaching either threshold (and stoppage of MDA) are depicted in these tables with and without inclusion of VC for both the 5-year TAS period as well as over a longer 20-year term following the breaching of these thresholds. The results demonstrate, firstly, as expected, that timelines to reach the 1% mf theshold will increase with endemicity but decreased in each of the three sites as annual treatments are replaced by biannual two-drug MDAs and by the annual administration of IDA irrespective of whether VC is included or not (Table 1). Thus, while annual single-drug MDA interventions (with or without VC) took the longest times to reach the 1% mf prevalence target among the present drug interventions, this threshold was reached earlier in the low transmission village of Piapung (5 years) compared to the need for 9 years of annual mass treatments required for Dozanso with the same IVM+ALB regimen. Biannual MDA, however, is predicted to be the most effective strategy for achieving the 1% mf threshold in the present villages, given that it takes only 1 (Piapung) to 4 years (Dozanso) compared to the 2–5 years required by annual IDA to cross this threshold in the same villages (Table 1).

**Table 1:** Elimination and recrudescence probabilities for different MDA scenarios using the 1% mf prevalence threshold for each of the low (Piapung), medium (Missasso), and high-prevalence (Dozanso) sites.

	Piapung					Missaso					Dozanso				
		Elimination probability (%)		Recrudescence probability (%)			Elimination probability (%)		Recrudescence probability (%)			Elimination probability (%)		Recrudescence probability (%)	
Scenario	Time to cross threshold (year)	After 5 years	After 20 years	After 5 years	After 20 years	Time to cross threshold (year)	After 5 years	After 20 years	After 5 years	After 20 years	Time to cross threshold (year)	After 5 years	After 20 years	After 5 years	After 20 years
Annual MDA upto 1% mf	5	0	40	15	36	6	1	29	34	64	9	12	49	34	46
Annual MDA+VC upto 1% mf	5	1	40	15	36	6	1	35	33	57	9	16	57	28	40
Annual MDA upto 1% mf, endgame VC	5	1	100	1	0	6	1	100	0	0	9	15	100	0	0
Annual MDA upto 1% mf, then only VC for the next 5 years	5	1	68	1	9	6	1	49	0	34	9	15	63	0	30
Biannual MDA upto 1% mf	1	0	1	30	75	3	0	19	34	72	4	2	26	38	65
Biannual MDA+VC upto 1% mf	1	0	1	30	74	3	0	21	34	69	4	2	29	36	62
Biannual MDA upto 1% mf, endgame VC	1	0	100	3	0	3	1	100	0	0	4	2	100	0	0
Biannual MDA upto 1% mf, then only VC for the next 5 years	1	0	14	3	13	3	1	38	0	36	4	2	40	0	48
Annual IDA upto 1% mf	2	0	1	31	73	4	0	6	42	87	5	0	8	51	78
Annual IDA+VC upto 1% mf	2	0	2	30	71	4	0	7	40	85	5	0	12	51	78
Annual IDA upto 1% mf, endgame VC	2	0	100	2	0	4	0	100	0	8	5	0	100	0	0
Annual IDA upto 1% mf, then only VC for the next 5 years	2	0	19	2	13	4	0	25	0	52	5	0	25	0	61

**Table 2:** Elimination and recrudescence probabilities for different MDA scenarios using the site-specific 95% EP mf thresholds for Piapung, Missasso, and Dozanso.

	Piapung					Missasso					Dozanso				
		Elimination probability (%)		Recrudescence probability (%)			Elimination probability (%)		Recrudescence probability (%)			Elimination probability (%)		Recrudescence probability (%)	
Scenario	Time to cross threshold (year)	After 5 years	After 20 years	After 5 years	After 20 years	Time to cross threshold (year)	After 5 years	After 20 years	After 5 years	After 20 years	Time to cross threshold (year)	After 5 years	After 20 years	After 5 years	After 20 years
Annual MDA upto 95% EP	20	31	99	0	0	22	72	100	0	0	24	54	97	0	0
Annual MDA+VC upto 95% EP	14	27	98	1	1	19	71	97	0	3	20	57	98	1	2
Annual MDA upto 95% EP, endgame VC	20	31	100	0	0	22	72	100	0	0	24	54	97	0	0
Annual MDA upto 95% EP, then only VC for the next 5 years	20	31	100	0	0	22	72	100	0	0	24	54	97	0	0
Biannual MDA upto 95% EP	11	2	100	0	0	12	13	99	0	0	13	15	100	0	0
Biannual MDA+VC upto 95% EP	8	1	96	0	2	11	12	96	0	3	11	13	97	0	3
Biannual MDA upto 95% EP, endgame VC	11	2	100	0	0	12	13	100	0	0	13	15	100	0	0
Biannual MDA upto 95% EP, then only VC for the next 5 years	11	2	100	0	0	12	13	100	0	0	13	15	100	0	0
Annual IDA upto 95% EP	13	1	99	0	0	16	18	100	0	0	17	23	100	0	0
Annual IDA+VC upto 95% EP	10	0	98	1	1	13	18	100	0	0	15	23	100	0	0
Annual IDA upto 95% EP, endgame VC	13	1	100	0	0	16	18	100	0	0	17	23	100	0	0
Annual IDA upto 95% EP, then only VC for the next 5 years	13	1	100	0	0	16	18	100	0	0	17	23	100	0	0

The second finding arising from the results depicted in Table 1 is that using annual MDAs without the inclusion of long-term VC, counterintuitively resulted in greater elimination (between 1 and 16% probability of elimination after 5 years and between 29 and 68% probability of elimination after 20 years) and lower recrudescence (between 0 and 34% recrudescence probabilities after 5 years, and between 0% to 64% recrudescence probabilities after 20 years) in these 3 villages compared to the application of the corresponding biannual MDA and IDA interventions (elimination probabilities generally between 0 and 29% with corresponding recrudescence probabilities reaching generally >30–87%) following the achievement of the 1% mf threshold.

The results show that combining VC with MDA from the start until the 1% mf threshold is reached did not overly improve the results obtained with the use of MDA alone, irrespective of the MDA strategy; however, including VC over the long term (i.e., for 20 years after the achievement of the 1% mf threshold by MDA) had an dramatic effect in increasing the probability of elimination (to 100%) and reducing the corresponding probability of recrudescence (to as low as 0 to 8%) depending on site and MDA strategy. Including VC after the achievement of the 1% mf threshold by MDA for over the 5-year TAS period alone, however, resulted in significantly lower elimination probabilities compared to when it was included over the longer 20-year period for all interventions. It did not also improve on using drug interventions alone for achieving transmission elimination over this short period (Table 1). As a result of this, all estimated recrudescence probabilities were found to be higher for the 5-year TAS period compared to the corresponding values calculated for the longer-term 20-year period, highlighting the need for long-term continuation with VC beyond the 5-year TAS period to ensure the breakage of parasite transmission if the 1% mf prevalence is used as the LF elimination target.

The corresponding results on timelines to extinction and probabilities of elimination and recrudescence using the site-specific 95% EP mf thresholds as the extinction target in each village is shown in Table 2. These show that using these thresholds will first of all significantly lengthen the durations of interventions required to break parasite transmission. Thus, while using annual single MDAs will take between 14 and 24 years to cross these thresholds in the three example villages—as compared to just 5–9 years it would take for this intervention to cross the 1% mf threshold in the same villages depending on site and inclusion of VC (Table 1)—this will be reduced to between 8–13 and 10–17 years of treatments required when the corresponding biannual and annual IDA MDA strategies are used in these villages (in comparison with just the 1 to 4 and 2 to 5 years required by these interventions to reach the 1% mf threshold). Adding VC to each MDA strategy from the beginning, by contrast to the results arising from using the 1% mf threshold, however, despite still taking longer, will result in reducing the overall durations required (by at least 2–6 years depending on strategy and site) to reach the 95% EP threshold (Table 2).

The estimated probabilities of elimination and recrudescence depicted in Table 2, on the other hand, highlight the major benefit of using the 95% EP threshold as targets for LF elimination programs, viz. that once these thresholds are breached by an intervention, it will inevitably lead to interruption of transmission but again as with the results shown in Table 1 for using the 1% mf prevalence target, significantly high probabilities of elimination (> 96%) and close to zero probabilities of infection recrudescence will occur only over the longer 20-year period irrespective of site or type of MDA-based strategy (Table 2). Another interesting, but perhaps expected finding, is that simply using MDA alone for achieving the 95% EP threshold, in contrast to using the 1% mf target, is also adequate for accomplishing transmission elimination as well as reducing the probability of infection recrudescence to close to zero over the long-term irrespective of the vagaries of site conditions and whether VC is included or not (compare Tables 1 and 2). Increasing the frequency of MDA (to biannual treatments) and use of annual IDA, however, again resulted in lower elimination and higher recrudescence probabilities following the 5-year TAS period irrespective of whether VC was included or not when these mf prevalence 95% EP targets are used (Table 2).

Table 3 further provides estimates of the times required to breach the corresponding L3 95% EP threshold in each of the three settings. These show that compared to crossing the 95% EP mf threshold, the time to cross each L3 threshold is faster, with the times being approximately half that taken to cross the mf threshold applicable in each setting (Table 2). Despite this, as in the case of the mf threshold, high probabilities of transmission elimination were achieved in each site only after a significantly long period following the breaching of even these L3 thresholds although this again was achieved approximately over half the time (10–15 years) that took when the corresponding mf thresholds are used as targets. The predicted times for crossing the L3 threshold also demonstrate why elimination probabilities overall were estimated to be low when the 1% mf prevalence threshold is used as the target for indicating LF elimination in these settings, viz. that the latter threshold will be inevitably achieved much earlier and MDAs stopped before the L3 threshold is breached (compare Tables 1 and 3).

**Table 3:** Elimination probabilities predicted for different MDA scenarios over various time periods following the stoppage of MDA using the 95% EP mf thresholds estimated for one low (Piapung), one med (Missasso), and one high prev (Dozanso) site.

	Piapung						Missaso						Dozanso						
			Elimination probability (%)						Elimination probability (%)							Elimination probability (%)			
Scenario	Time to cross mf threshold (year)	Time to cross L3 threshold (year)	After 5 years	After 10 years	After 15 years	After 20 years	Time to cross mf threshold (year)	Time to cross L3 threshold (year)	After 5 years	After 10 years	After 15 years	After 20 years	Time to cross mf threshold (year)		Time to cross L3 threshold (year)	After 5 years	After 10 years	After 15 years	After 20 years
Annual MDA upto 1% mf	20	10	31	98	99	99	22	15	72	99	99	100	24		16	54	96	97	97
Annual MDA+VC upto 1% mf	14	7	27	90	97	98	19	10	71	96	97	97	20		12	57	97	98	98
Annual MDA upto 1% mf, endgame VC	20	10	31	98	100	100	22	15	72	100	100	100	24		16	54	96	97	97
Annual MDA upto 1% mf, then only VC for the next 5 years	20	10	31	98	100	100	22	15	72	99	100	100	24		16	54	96	97	97
Biannual MDA upto 1% mf	11	5	2	76	99	100	12	8	13	94	99	99	13		9	15	90	100	100
Biannual MDA+VC upto 1% mf	8	3	1	49	88	96	11	5	12	62	84	96	11		6	13	60	83	97
Biannual MDA upto 1% mf, endgame VC	11	5	2	77	100	100	12	8	13	95	100	100	13		9	15	90	100	100
Biannual MDA upto 1% mf, then only VC for the next 5 years	11	5	2	77	99	100	12	8	13	95	100	100	13		9	15	90	100	100
Annual IDA upto 1% mf	13	7	1	82	99	99	16	10	18	99	100	100	17		11	23	95	100	100
Annual IDA+VC upto 1% mf	10	5	0	57	93	98	14	7	18	97	99	100	15		8	23	91	100	100
Annual IDA upto 1% mf, endgame VC	13	7	1	84	100	100	16	10	18	99	100	100	17		11	23	97	100	100
Annual IDA upto 1% mf, then only VC for the next 5 years	13	7	1	83	99	100	16	10	18	99	100	100	17		11	23	97	100	100

Inclusion of VC with MDA from the beginning helped to reduce the time to reach the site-specific 95% EP mf thresholds, but it was found not to add any additional benefit to increasing the probability for achieving transmission elimination or reducing the probability of recrudescence over that accomplished by MDA alone (carried out until the 95% EP mf thresholds are reached) for either the 5-year TAS period or over the longer 20-year period (Table 2). Applying VC during 5 years (TAS period) after achievement of the 95% EP mf threshold by MDA alone will also result in almost the same benefit in increasing the probability for achieving transmission elimination or reducing the probability of recrudescence as that accomplished by carrying out either MDA alone or by implementing longer-term supplementary VC (Table 2).

### Dynamics of LF prevalence recrudescence arising from applying the WHO and 95% EP mf thresholds

**Fig. 3:**
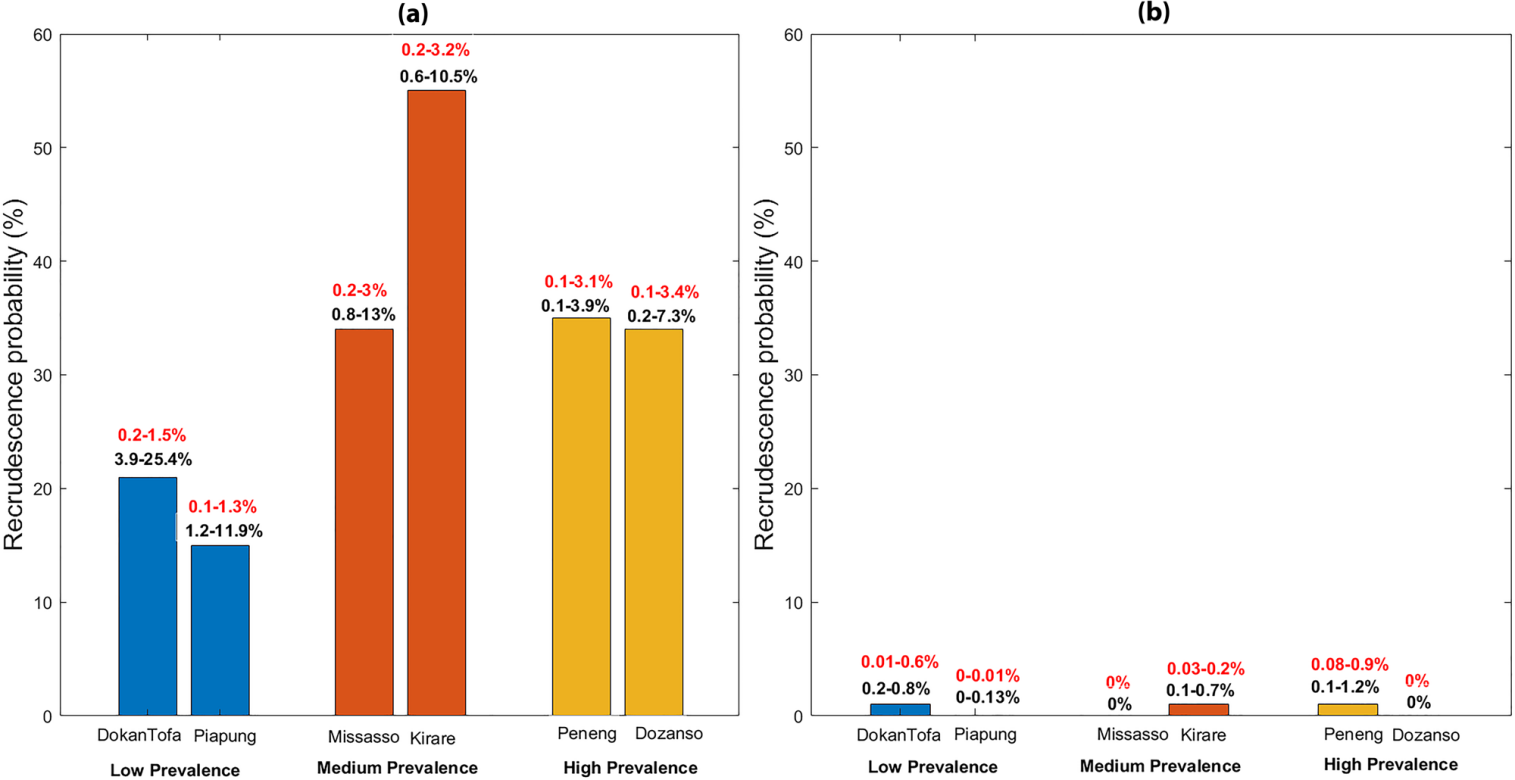
Bar diagram of recrudescence probability for two low-prevalence (DokanTofa, Piapung), two medium-prevalence (Missasso, Kirare), and two high-prevalence (Peneng, Dozanso) study sites. **a** After 5 years of stopping annual MDA following breaching of the 1% mf threshold, and **b** after 5 years of stopping annual MDA following breaching of the 95% EP threshold as shown in Tables 1 and 2 and Supplementary Tables 4 and 5. Figures in red above each bar indicate the predicted ranges in mf prevalence while figures in black above each bar indicate the mf prevalence predicted as a proportion of baseline mf prevalence.

Figure 3 summarizes the recrudescence probabilities along with the actual average levels of mf prevalence (red text over bars) and mf prevalence as a proportion of baseline prevalence (black text over bars) predicted in each of our six study sites by the end of the 5-year TAS period as a result of applying annual MDA alone for meeting either the WHO 1% mf target (Fig. 3a) or the estimated site-specific 95% EP mf thresholds (Fig. 3b; see also Tables 1 and 2). The results show that not only will the predicted recrudescence probabilities reach markedly higher levels when interventions are stopped once the 1% mf target is achieved compared to when the 95% EP thresholds are crossed, but that the reinfection prevalence attained will also be significantly higher in the former scenario (mf prevalences reaching between 0.1 and 3% when the WHO target is used compared to just 0–0.01% in the case of using the 95% EP mf thresholds).

**Fig. 4:**
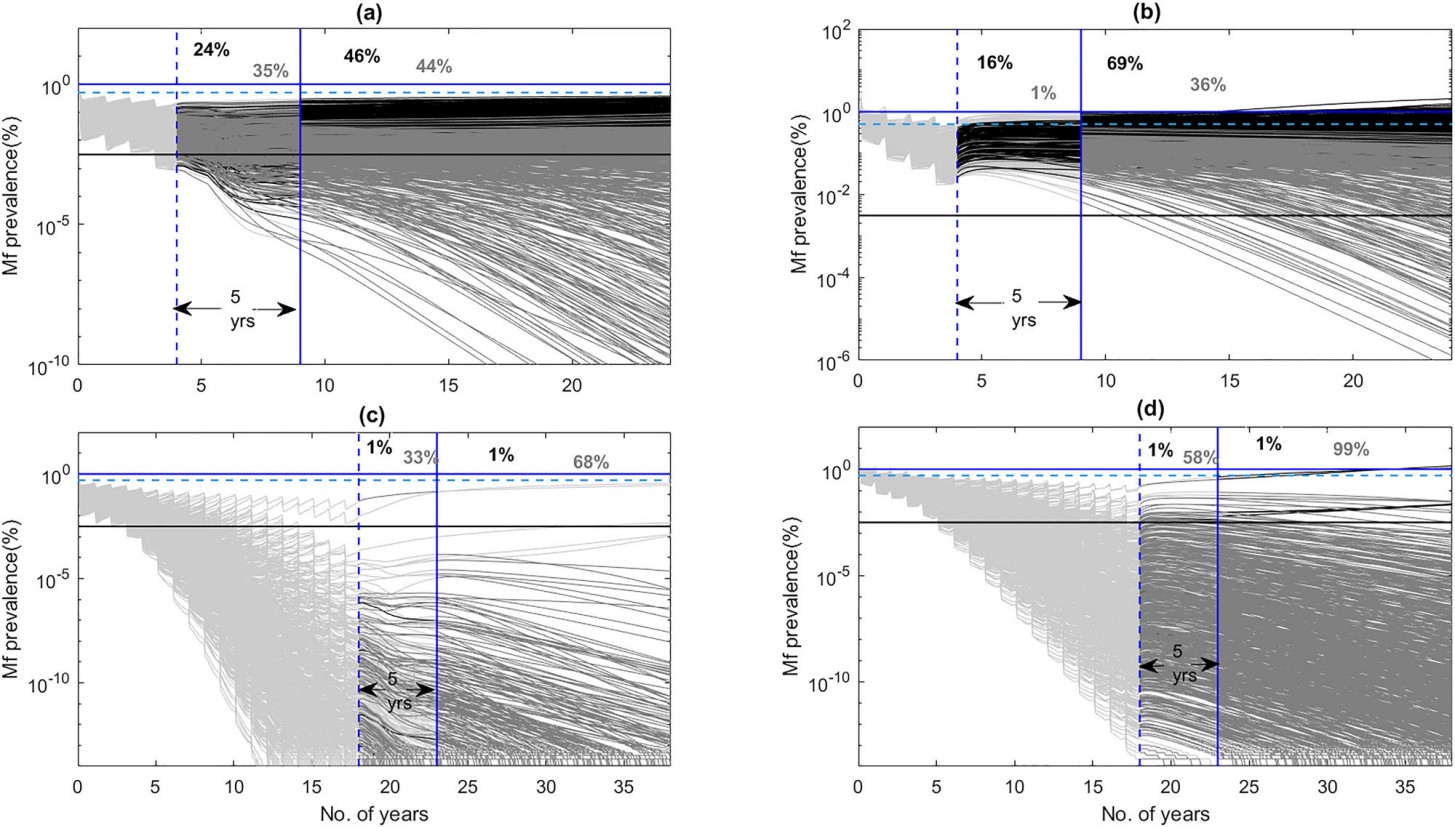
Impact of annual MDA in DokanTofa, Nigeria. **a** When annual MDA was continued to cross the 1% community mf threshold on mf prevalence in 6–7 years old children, **b** when annual MDA was continued to cross 1% mf threshold in the 40–70 years old population, **c** when annual MDA was continued to cross the corresponding 95% EP mf threshold in 6–7 years old children, and **d** when annual MDA was continued to cross the 95% EP mf threshold in the 40–70 years old population. Light gray lines indicate the model predictions with colors changing to black when trajectories begin to ascend and to dark gray when they move toward elimination. Numbers given in the black and dark gray colors indicate the probabilities (as percentages) of infection recrudescence and elimination respectively. Blue solid, blue dotted, and black solid horizontal lines indicate the 1% mf threshold, 0.5% mf threshold and model predicted 95% EP mf threshold, respectively, whereas blue dotted and blue solid vertical lines represent the time periods for crossing the 1% mf threshold and the 5 year post-MDA stoppage duration (the TAS period), respectively.

Figure 4 provides an example of the temporal changes predicted for mf prevalence arising from the use of the WHO 1% mf target versus the deployment of the 95% EP mf thresholds in one of the present study villages, and highlights a major age-related negative consequence related to the use of the currently TAS recommended target for monitoring LF transmission elimination. The mf predictions depicted in the figure are those obtained from the application of annual MDA in the village of DokanTofa, Nigeria (Supplementary Table 2), and display the parasite elimination/recrudescence rates obtained in the post-TAS 6–7-year-old child survey population (Fig. 4a, c) compared to those predicted for these metrics in the older 40–70-year-old population (Fig. 4b, d). The upper panel of the figure (Fig. 4a, b) illustrates the findings from the use of the WHO target, while the bottom panel (Fig. 4c, d) highlights the corresponding predictions resulting from the use of the 95% EP mf threshold applicable to this site. These results highlight two key outcomes. First, using the WHO threshold will lead to higher recrudescence rates (and conversely lower elimination probabilities) in both the 6–7 year-old child age (24% for a 5-year TAS period; 46% for longer term 20-year period) as well as in the 40–70-year-old (16% for 5-year TAS period; 69% for longer-term 20-year period) populations (Fig. 4a, b) compared to strikingly lower probabilities of recrudescence estimated for these respective populations when the 95% EP mf targets are used (Fig. 4c, d). The second significant result is that in the case of using the 1% mf threshold, the estimated recrudescence will occur even though over the 5-year TAS period, mf prevalence in the 6–7-year-old child population (which represents the post-TAS monitoring population) will be lower than both the 1% (TAS target) and 0.5% mf prevalence (the proposed post-TAS target for mf) levels (Fig. 4a). Note that infection levels will reach above both these TAS and post-TAS levels by year 5 post-intervention and indeed continue to increase in the older population with time (Fig. 4b), indicating that despite infection detected to be lower than the post-TAS 0.5% mf prevalence threshold in the target child age survey group, the transmission will continue in the community owing to the continued presence of significant infection prevalences in the (unmonitored) older age populations. By contrast, the predicted recrudescence rates will be insignificant and levels of LF infection will decline to zero for both these populations once the baseline community prevalence is reduced to below the site-specific 95% EP threshold and MDA is discontinued (Fig. 4c, d). Similar results were found for all other study sites.

### Transient dynamics and slow regime change

**Fig. 5:**
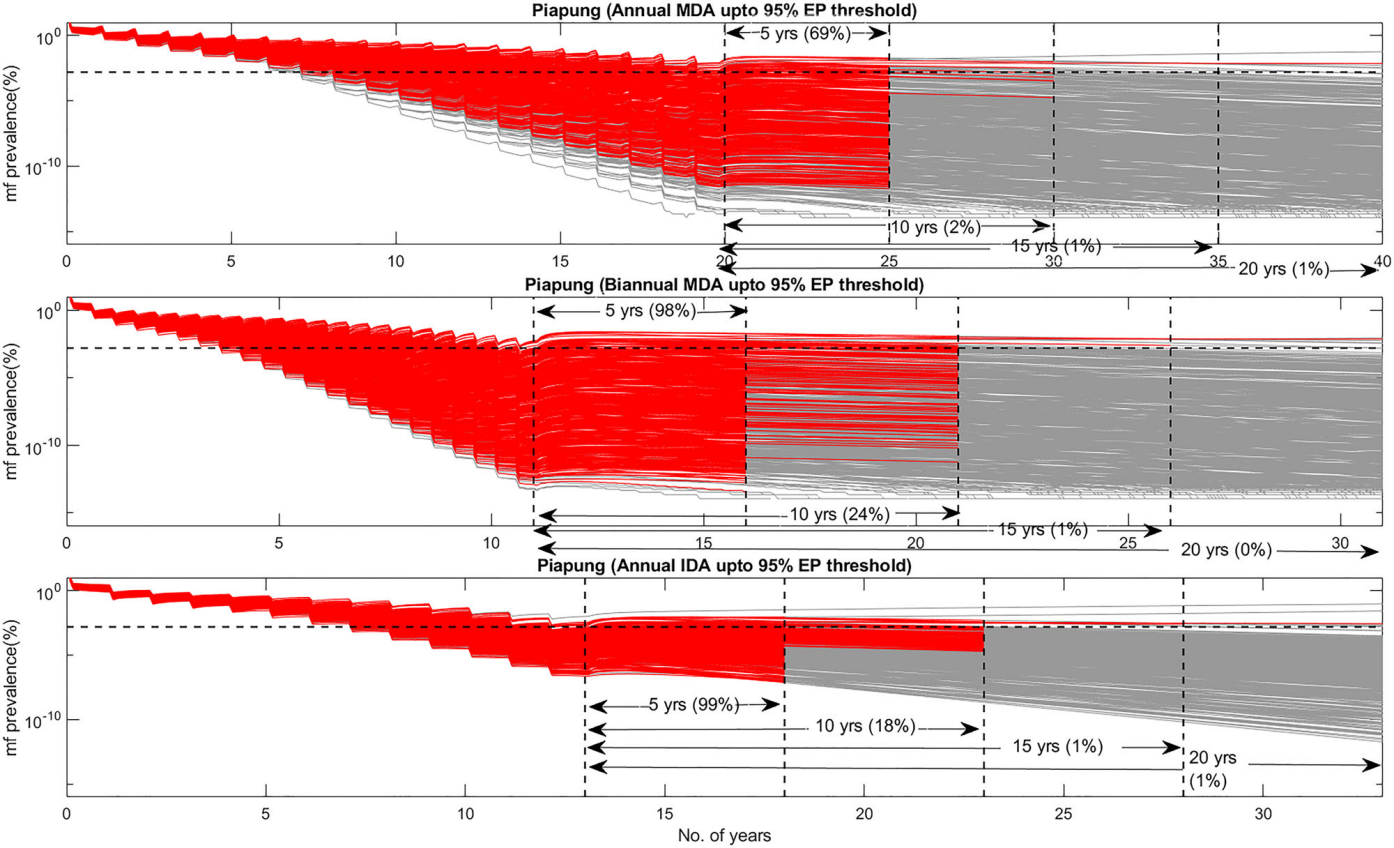
Predicted transient dynamics in mf prevalence using annual MDA, biannual MDA, and annual IDA and the 95% mf prevalence EP threshold for Piapung. Red curves indicate the model fits with transient behavior (curves having no significant positive or negative slopes) whereas the gray curves indicate the model fits having significant positive or negative slopes for different time periods (5, 10, 15, and 20 years) after stopping MDA following breaching of the 95% EP mf threshold. The figures in percentage show the percentage of the curves describing transient behaviors.

The foregoing results highlight that the transition to LF extinction may take a long time to occur following either the breaching of transmission breakage thresholds (irrespective if these are mf or L3 thresholds) or reduction of infection levels close to such thresholds. This suggests that the shift to extinction after these thresholds are exceeded will not be abrupt but will unfold over a long period of time. Figure 5 illustrates this slow, long-lasting, shift to the extinction of infection following the passing of the 95% EP mf prevalence threshold estimated for Piapung. The results also demonstrate that this slow regime shift is primarily driven by the transient or non-equilibrium behavior that evolves immediately following the breaching of the EP threshold (as depicted by the red curves), which dissipates only over a long period of time resulting in a progressively greater fraction of model trajectories (gray curves) eventually declining to the zero state. Similar transient results are also obtained post-crossing the L3 thresholds shown in Table 3 (Supplementary Fig. 1).

Table 3 shows a more detailed breakdown of the predicted probabilities of elimination in the three villages of Piapung, Missano, and Dozanso following the breaching of their respective 95% mf prevalence thresholds by the various interventions modeled in this work. Three outcomes are immediately apparent. First, in concert with the results shown in Table 2, the application of annual MDA will generally allow the dissipation of transient behavior and hence achievement of high probabilities of elimination (>95%) earlier (10 years after breaching of the 95% EP thresholds except in the case of inclusion of VC with MDA up to meeting the threshold) compared to the use of the corresponding biannual and IDA MDA regimes in each site (with transient behavior and achievement of elimination occurring only after 15 years following the passing of this threshold). The second outcome of interest is that including VC into MDA programs from the beginning, while allowing the achievement of the 95% EP thresholds earlier in each site, will extend the transient period and push out the time to achievement of transmission elimination irrespective of MDA regimen (Table 3). Together, thus, these results show that the earlier the EP thresholds are reached by the more frequent or intense MDA programs (the biannual and IDA interventions as well as in the case of including VC into MDA programs from the beginning), the longer the period of transient regimes produced and hence times to extinction following the passing of system thresholds. The results for L3 prevalence following the crossing of the 95% mf threshold shown in Supplementary Fig. 1 (shown for Piapung) suggest that this counterintuitive finding may be an outcome of the more intensive but shorter-term interventions interacting with the faster feedback loops governing L3 infection in the vector population resulting in the production and sustenance of longer operating larval transient states as annual MDA is replaced by biannual MDA or annual IDA.

Nonetheless, it is clear that if the total numbers of years predicted for achieving high probabilities of elimination are taken into account (i.e., both the times to reach the EP threshold and the time to high probability (>95%) of extinction), then the results shown in Table 3 suggests that biannual MDA and IDA may allow the achievement of parasite elimination earlier (by between 4 and 6 years depending on regimen and baseline endemicity) compared to using the corresponding annual MDA-based interventions. This is primarily because such more intensive interventions allow the passing of the estimated 95% EP mf prevalence thresholds significantly earlier than in the case of the annual MDA regimens (Tables 2 and 3).

### Assessing the validity of model forecasts using field data

**Fig. 6:**
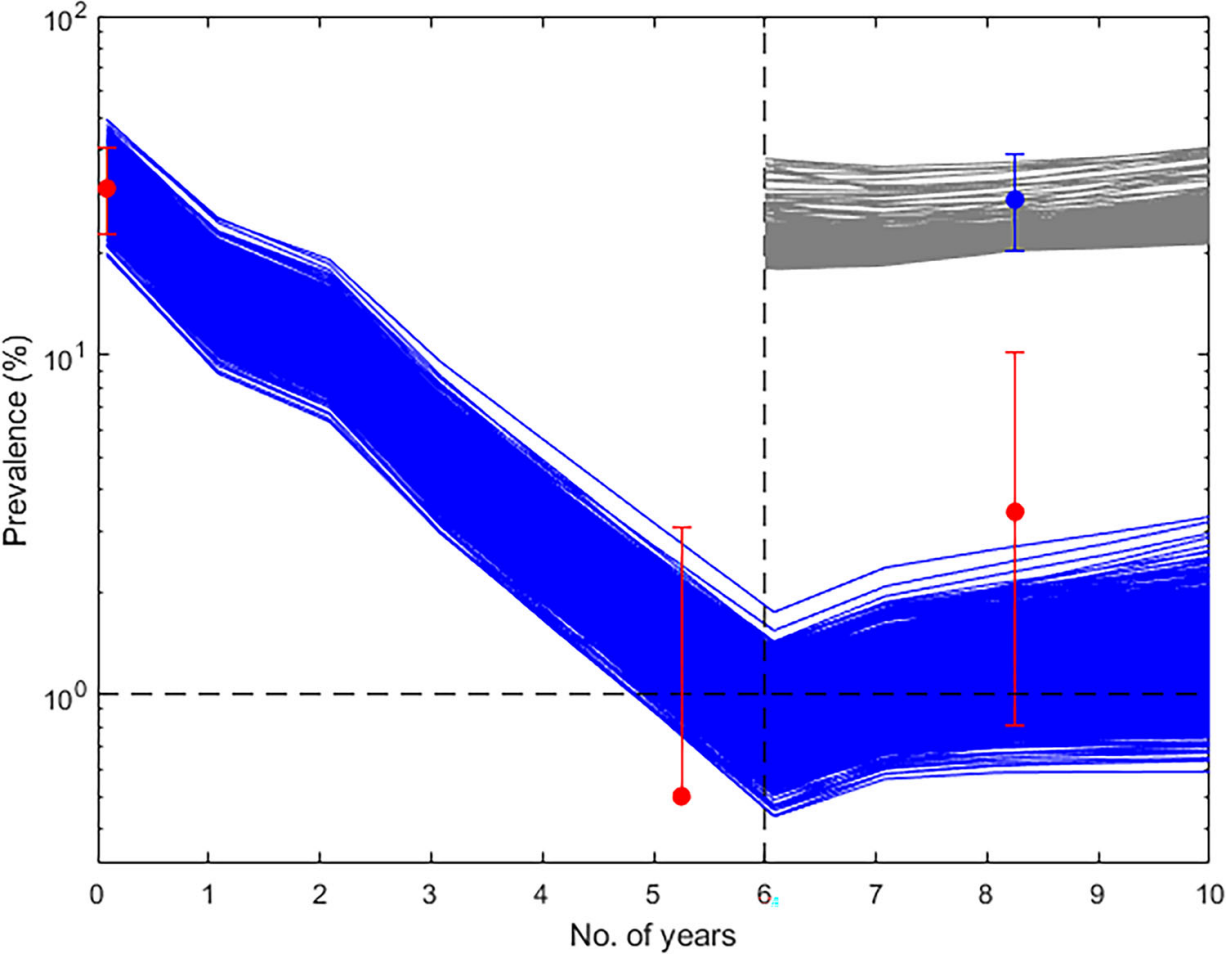
Model-predicted impact of MDA on mf prevalence during seven rounds of MDA and after stopping MDA, and on CFA prevalence at the post-MDA survey in year 2008 for Leogane commune in Haiti. Blue lines indicate the predicted impact of the interventions on mf prevalence whereas gray lines indicate the predicted impact of the interventions on CFA prevalence. Red dots indicate field-observed data points for mf while the blue dot is the corresponding data point observed for CFA. Horizontal and vertical black dotted lines indicate the WHO recommended 1% mf threshold and the time point of stopping MDA, respectively. Error bars indicate the 95% confidence intervals of the data points.

Field data to corroborate the above model predictions are still scarce, but data from two studies allowed evaluation of the impact of adding vector control to MDA programs as a measure for reducing infection recrudescence after stopping MDAs once prevalences are reduced to near 1% mf prevalence^18^, and if this would indeed lead to sustained interruption of transmission once crossed and MDAs are stopped. By contrast, surveillance data on the impact of annual MDA using DEC+ALB assembled from the commune of Leogane in Haiti allowed the direct investigation of whether using the WHO recommended 1% mf threshold is sufficient to bring about LF transmission interruption and hence support the stopping of intervention^13,19–22^. In the latter setting, seven rounds of MDA were delivered starting from year 2000 with variable coverages and compliances and mf prevalence was reduced below the 1% threshold by the 6th year of treatments (in 2005); however, a subsequent survey carried out in whole population in year 2008 indicated that transmission was still ongoing in the community despite the apparent success of the applied MDAs^13^. Figure 6 depicts both the changes in mf prevalence in the community until year 7 post-MDA and the prevalences for both CFA (blue points/lines) and mf (red points/lines) at the post-MDA survey in year 2008. It also portrays the predictions of our model calibrated to the baseline mf data for the annual MDA interventions carried out in the commune (by taking also into account the actual coverages attained annually). These results provide important empirical evidence indicating that the WHO 1% mf threshold has not led to interruption of LF transmission in this setting, and indeed that, as the model forecasts show (and mimicking the theoretical results above), the use of this threshold will instead result in significant corresponding resurgences of infection if its breaching forms the basis for deciding to stop further interventions.

**Fig. 7:**
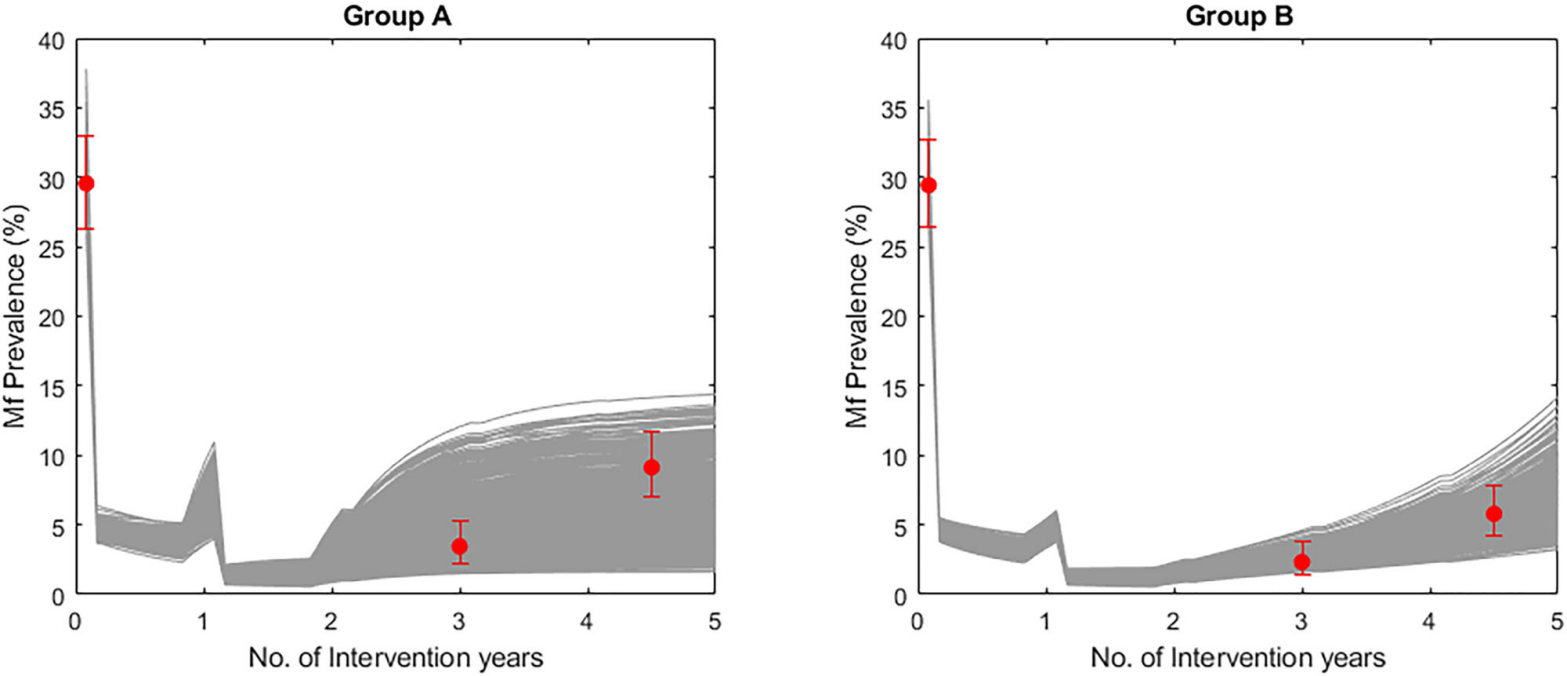
Model-predicted impact of MDA alone (Group A villages) and MDA supplemented with VC throughout the intervention period (Group B villages) on mf prevalence. Gray lines indicate the predicted impact of the interventions on mf prevalence and red dots indicate field data points. To implement simulations of the impact of integrated vector management (IVM) interventions carried out in Group B we fitted a segmented exponential function for monthly biting rate (MBR): *MBR* *=* *MBR*_*0*_*exp[a*_*1*_*t]*, with a_1_<0 for all t when VC is ON, otherwise a_1_>0^44^. Here we took a_1_ = −5 for our model simulations. Error bars indicate the 95% confidence intervals of the data points.

In the study by Sunish et al.^18^ on the other hand, one group of villages received two annual MDAs of DEC+IVM), while Group B villages received the same MDA schedule in combination with VC, chiefly using expanded polystyrene beads (EBP), biolarvicide and larvivorous fish (Supplementary Table 6). MDAs were stopped after two annual treatments and the communities were resurveyed for infection levels three years after the stoppage of MDAs in both groups of villages. VC, however, continued in the Group B villages following cessation of MDA. The results from the post-treatment surveys showed that VC preserved the effects of MDA while resurgences occurred in Group A villages (Supplementary Table 6). Overall, continuing with vector control suppressed the mf prevalence attained three years following stoppage of MDA in the Group B villages by up to 55.8% compared to the mf prevalence reached in Group A (Supplementary Table 6). Figure 7 shows the corresponding model predictions in comparison with observed data, and highlights that the theoretical findings described above regarding the impact of using supplementary vector control for both retaining the gains of MDA and arresting LF recrudescence may indeed operate in the field.

## Discussion

Infection thresholds that mark the boundary between the endemic and the extinct states of a parasitic transmission system increasingly form a core foundation of disease eradication policies^23–27^. This is because for these programs they are deemed to present the target measures that can be acted upon to facilitate the breaking of parasite transmission^17,26,27^. This concept also forms the mainstay of WHO’s LF eradication program, in which it is proposed that reducing endemic community-wide infection prevalences to below 1% mf or 2% CFA prevalence first by MDA before switching to follow-up surveys over a 5-year period to determine if infection levels remained below half these values in children aged between 6 and 7 years is sufficient to ascertain if transmission has been broken and hence allow the ending of interventions in a setting^4^. The decision rule for this TAS protocol is that if both the above criteria are met, then it can be assumed that parasite transmission has been broken, or else MDA is to be resumed. Yet, despite the steady roll out of this phased assessment strategy since 2011 in almost all endemic countries, empirical evidence to determine if meeting the proposed 1% mf or 2% CFA threshold do indeed lead to breakage of local transmission everywhere is scarce. In fact, increasing field studies have shown that passing these thresholds may not result in the cessation of transmission over the 5-year TAS period in every endemic community^6–8^, casting considerable doubt regarding the validity of using the proposed thresholds as globally reliable measures to guide LF intervention-stopping decisions.

Here, we have leveraged the ability of a deterministic model of LF transmission fitted using a Monte Carlo approach to data from 6 representative endemic communities to evaluate the implications of using the above WHO TAS criteria for determining the interruption of parasite transmission in a treated community. This was done by firstly estimating the values of the LF elimination thresholds that may apply in each locality followed by characterizing the resulting extinction dynamics that occurs subsequent to the passing of either the WHO or the thresholds estimated by our model. We also use simulations of the outcomes of applying various MDA regiments with and without vector control to determine the best strategy for achieving the sustained elimination of LF transmission when using either threshold as the intervention-stopping target. Given anticipated difficulties in measuring the model-based thresholds reported here, we also used our simulation results to provide new information regarding the intervention strategies that could allow or improve the probability of achieving LF elimination using more measurable criteria, such as that the one set by the WHO.

We began our investigation by first quantifying the values of the mf prevalence extinction thresholds likely to occur in each of the present study settings using the models that best fitted their baseline age-prevalence data. We note in passing here that given we identify and use 500 best-fitting models reflecting parameter uncertainty and variability from each site to make these calculations (see “Methods”), this will, and resulted, in the generation of a cloud of extinction thresholds rather than a single point estimate for a site^28^. While each of the estimated thresholds in this ensemble when crossed can thus result in transmission interruption, we present and use those thresholds the passing of which in each site will give rise to at least a 95% chance of transmission extinction (see method of estimation given in “Methods”^29,30^) in our analysis throughout. This is to reflect the management preference in disease eradication programs for averting the risk of making a wrong stopping decision arising from using a higher valued threshold selected from among the ensemble of thresholds estimated for a setting which would result in lower probabilities of elimination, compared to employing lower-valued thresholds from among the set the passing of which would result in higher elimination probabilities. As we have shown previously^9,10^, in contrast to the WHO recommended 1% mf prevalence threshold to be applied commonly in all settings, these 95% EP mf threshold values predicted by the locality-specific LF models will invariably be significantly much lower than the currently set TAS threshold (Supplementary Table 5). They will also be lower in their values when estimated at the prevailing, unperturbed, annual vector biting rate (ABR) compared to when quantified at the vector threshold biting rate (TBR)^9,10^. Importantly, these thresholds will also be invariably variable between sites (Supplementary Table 5), casting further reservations on the recommendation for using a single globally applicable threshold in the current TAS surveillance strategy^9,14,31^.

Nevertheless, the values estimated for the 95% EP mf prevalence thresholds (Supplementary Table 5) indicate that these could be numerically very low, implying that for small populations (<10,000 individuals) the estimated values would equate zero cases in practice. This would make these thresholds difficult to measure under field conditions, with major implications for efforts to determine when LF transmission interruption may have been achieved. A key question therefore is whether there is field support for the existence of these low-valued thresholds as determined by our essentially deterministic models. We offer the following lines of evidence to denote that LF thresholds in nature may indeed be closer to the model estimates shown in Supplementary Table 5 than to the 1% mf prevalence threshold proposed by the WHO. First, the values of the linear aggregation parameters governing the distribution of infection in each of the present populations as estimated by our model show the operation of very high degrees of infection clustering in each setting (Supplementary Table 5). Such high levels of heterogeneity can act to decrease the values of parasite transmission thresholds^31,32^. Second, as noted there is growing field evidence that passing the 1% mf prevalence TAS threshold is no guarantee for bringing about transmission elimination^6–8^. Our simulations of the impact of annual MDA using DEC+ ALB and the corresponding data from the commune of Leogane in Haiti (Fig. 6) further highlight this possibility, including the potential for significant resurgence of infection if arbitrary thresholds (such as the 1% mf prevalence threshold) unrelated to the underlying extinction dynamics are used. Finally, we have previously demonstrated field support from Papua New Guinean study villages for predictions made by our model for vector biting rate thresholds, whereby in those villages that achieved a high probability of crossing their predicted monthly biting rate (MBR) threshold by means of vector control we detected zero infections in subsequently sampled mosquito populations^29^. Thus, although stochastic events especially in small populations might indicate the possibility for the existence of higher thresholds and subsequently low resurgence rates post interventions^12,33^, the above suggests that at least for moderately large populations, LF thresholds are likely to low-valued although ultimately field studies employing novel survey approaches focused on minimizing the needed field sample sizes (such as those afforded by sequential sampling methods^30^) will be required to test this proposition empirically.

The outcomes of using the WHO versus the model-estimated 95% EP mf prevalence threshold targets for determining the achievement of parasite elimination and recrudescence were then determined over the short-term TAS period (after 5 years of MDA stoppage) as a result of carrying out the annual MDA interventions implemented in each of the present study sites (see Supplementary Tables 4 and 5). We note firstly here that while using the 1% mf threshold set by WHO would invariably lead to shorter durations of annual MDA required for its breaching in each site, the calculated probabilities of transmission interruption achieved would be low or moderate ranging from 1 to 41% with the subsequent probability of recrudescence ranging from 15% to as high as 55% over the 5-year post-MDA (ie. the putative TAS) period (Supplementary Table 4). Figure 3 further illustrates that the range in prevalence values that may be expected following intervention stoppage after crossing the 1% mf threshold could also be wide and reach high levels (between 0.1 and 2.9% among the sites) just after the 5-year period, suggesting that the present sites would most likely fail the current TAS criteria (sustained suppression of mf prevalence <1% in a community) over the period of TAS survey bouts). By contrast, while it will take significantly and variably longer (up to 19 years of annual MDA depending on baseline mf prevalence) to cross the site-specific 95% EP mf thresholds, our results show that high probabilities of parasite elimination (31–98%) and consequently very low probabilities of infection recrudescence (close to zero) will be achieved over the 5-year TAS period if these thresholds arising from the learned LF system dynamics in a site are used as MDA elimination targets in the present sites (Supplementary Table 5). The data presented in Fig. 3b further shows that the infection prevalences achieved following the stoppage of interventions over the 5-year recommended TAS surveillance period will also be low and largely around or significantly below 1% mf prevalence (*P* <0.01%), meaning that all the present sites will likely pass the TAS criteria for stopping interventions if the model-estimated 95% EP mf threshold for each site is used as the target for signifying the achievement of LF transmission elimination.

The timelines estimated to cross L3 prevalence EP thresholds provided for three of the present villages in Table 3 provide an answer to why using the crossing of 95% EP mf prevalence thresholds as a basis for stopping MDA leads to higher elimination probability compared to when the WHO 1% mf prevalence threshold is used for this purpose. These results show that as the 1% mf prevalence threshold will be invariably breached by annual MDA much before the L3 threshold is crossed (before at least 5 years), drug interventions will be stopped even as substantive transmission will be ongoing in a setting. By comparison, the MDA stopping decision-based on crossing the 95% mf prevalence will occur at least 5 years after the corresponding L3 thresholds are breached (Table 3), which would increase the probability for achieving transmission interruption. Nevertheless, two features of the calculated elimination probabilities shown in Supplementary Table 5 include the fact that in spite of the use of the 95% mf prevalence thresholds, the resulting probability of elimination can be as low as 31% after 5 years post-stoppage of interventions (ie. over the proposed WHO TAS period) with this probability generally increasing as annual MDA cycles are prolonged.

Our scenario-based simulations of the relative impacts of currently applied and proposed MDA-based strategies with and without VC on parasite elimination and suppression of infection recrudescence when either the WHO set 1% mf threshold or the site-specific 95% EP threshold is used have provided intriguing answers to the above findings, whilst also furnishing further insights on the choice of thresholds and interventions for bringing about sustainable LF transmission elimination. A first major finding in this regard is that while switching to biannual MDA and annual IDA with or without VC can significantly reduce the number of years of intervention required to cross the 1% mf prevalence threshold in comparison with the implementation of annual single MDA alone in each site, the probabilities of elimination eventually achieved long-term (20 years post-stoppage of MDAs) by meeting this threshold by the longer duration annual MDA was higher than that obtained from the application of the relatively faster-acting biannual MDA or annual IDA alone strategies in these sites (Table 1). The converse was true for the calculated recrudescence probabilities with values reaching as high as 75% in the low-prevalence sites when biannual MDA or IDA alone were used. Elimination probabilities and recrudescence probabilities were both comparatively lower for each of these MDA strategies when assessed after 5 years post-stoppage of MDA (Table 1).

By contrast, while the calculated elimination probabilities were again significantly lower after 5 years post-stoppage of MDA when the corresponding 95% mf prevalence EP thresholds were used (up to 72%), these were significantly elevated 20 years after stopping of the modeled MDAs (>95%) in all settings (Table 2). A difference was that when the 95% EP thresholds are used as MDA stopping targets, the probabilities of recrudescence by comparison with the results obtained using the 1% mf threshold were close to 0 over both the short 5-year and long 20-year post-intervention stoppage periods (Table 2). However, as with the use of the 1% mf threshold, elimination probabilities obtained when using the 95% EP thresholds over the short assessment period of 5 years was higher as the duration of MDAs needed to cross these thresholds increased, meaning that biannual MDA and IDAs allowed the crossing of these thresholds earlier but resulted in lower elimination of transmission over this period (compared to the 20-year period when elimination probabilities reached levels as high as that achieved by the longer duration annual MDA strategy (Table 2)).

Taken together, the above findings hint that complex trade-offs could occur between the faster ability of these more effective drug regimens (biannual MDA or annual IDA) for hastening the crossing of either threshold balanced by their inability to shift endemic infection to the extinct state. Analysis of natural systems behavior to stessors may provide an explanation for this behavior^26,34^. Such work suggests that more frequent perturbations as generated by biannual MDAs or more intensified interventions (IDA or MDA with VC) can interact with the response time of a system to induce shorther or longer transient phases as thresholds are crossed until a new system state emerges. Indeed, a system with strong feedback loops may exist almost continuously in a transient state if there is frequent disturbance^34^. We indicate that a similar mechanism, in which the more frequent and larger changes induced by the interventions other than annual MDA are countered by the relatively longer living LF system (adult worms living up to 10 years) by the generation of a long-lasting transient period (as measured by flips in the signs of the slopes of the model trajectories close to or after crossing of thresholds (Fig. 5)), may underlie the lower effectiveness of the biannaul MDA and IDA interventions modeled here for affecting transmission elimination during the period immediately (5 years) post-stoppage of MDA. Note that in the case of LF, such induction of transient behavior may be explicitly driven by the action of the strong density-dependent infection feedback loops operating in the vector population that will increase L3 transmission relative to decreases in mf intensity due to drug treatments^11,23^ (see Supplementary Fig. 1).

Our analysis further shows that the transition to the extinct state following LF interventions may occur over a protracted period of time even when the 95% EP thresholds (whether connected with mf or L3 prevalences) are used as elimination targets (Table 2 and Table 3). Depending on the intervention strategy, high probabilities of transmission elimination (>95%) in this regard were obtained only 10 or 15 years after the passing of these thresholds (Table 3). This is an hitherto unsuspected finding, which reveals that contrary to current thinking abrupt population thresholds should not be expected for LF. Furthermore, as shown in Fig. 5, this slow gradual regime shift is characterized by very low levels of persistent infection, which will also make it be difficult to detect in the field. A second outcome is that this protracted transient period may also signify the likely occurrence of an extended risky period of time when LF regime shifts could be reversed to prevent the new alternate extinct state from eventuating^34^. This will allow a return to the original endemic state particularly if in-migration of infected individuals or vectors occurs in the absence of vector control. Further work on transient behaviors by the LF system, including the feature of critical slowing down^35^ requires study, but this result suggesting that annual MDA may have a greater impact in affecting higher elimination and lower recrudescence over the immediate period after the crossing of thresholds highlights the need for fully addressing the implications of intervention-induced extinction dynamics when choosing strategies and timeframes to affect and monitor parasite transmission interruption reliably^9,33^.

The model predictions regarding the impact of including VC into the MDA strategies we studied suggest that the outcomes may be complicated with the use of this integrated strategy for accelerating and sustaining LF elimination, suggesting the need to be appreciative of the subtleties in the use of this approach. Thus, while combining VC with MDA from the start until the 1% mf threshold is reached is shown not to improve either the time to cross this threshold or the elimination or recrudescence probabilities calculated compared to the use of MDA alone, irrespective of the MDA strategy modeled (Table 1), including VC after the achievement of this threshold by MDA had a dramatic effect in increasing the probability of elimination to 71–99% and reducing the corresponding probability of recrudescence (0–29%) irrespective of site or MDA strategy for the longer term 20-year period (Table 1). This is an unexpected finding and crucially implies that the WHO-set 1% mf threshold for stopping MDAs can be used in LF programs provided it is coupled with the inclusion of long-duration VC after this threshold is reached. This impact of long-term post-MDA inclusion of VC for ensuring parasite elimination and preventing infection recrudescence alongside the use of the 1% mf threshold furthermore was robust irrespective of variations in local transmission conditions (Table 1), implying as we have demonstrated previously^10^ that this strategy could also serve as a method for overcoming the observed between-site heterogeneity in the transmission of LF. These results indicate overall that national LF programs must now seriously consider integrating existing VC programs, such as the malaria bednets^36,37^, to supplement LF MDA programs if the currently used WHO infection elimination targets are continued to be used in LF elimination programs. Indeed, plans should be devised to incorporate such VC over the longer term even after TAS is passed (perhaps up to 10 years at least post-TAS) if we are to ensure that LF is sustainably eliminated if programs continue to use the present WHO targets.

Using the 95% EP threshold as estimated here will obviate this need but it will take longer cycles of MDA to breach these thresholds (between 8 and 24 years depending on MDA regimen and inclusion of VC), compared to using the 1% mf threshold (Tables 1, 2). However, this needs to be balanced by the need for long-term VC (perhaps upto 10–15 years post-MDA, depending on the intervention) to ensure that transmission interruption is sustained once the latter threshold is crossed. Note also that switching to biannual MDA or IDA will decrease the number of years drug treatments required to cross the 95% mf threshold significantly to between 11 and 17 years (Table 2) or similarly to the years of MDA required when using MDA plus long-term VC in the case of applying the 1% mf threshold as described above. This means that logistical or feasibility challenges of applying longer-term MDA versus pursuance of long-term VC in different settings should then dictate the choice of the usable elimination target by LF national programs. However, our modeling of the recrudescence dynamics of mf following the meeting of the 1% threshold indicates another important liability of using this, threshold, viz. that while mf prevalence might meet the TAS criteria of being lower than the 0.5% prevalence target in the 6–7-year-old TAS monitoring group during the 5-year TAS assessment period, it will both remain above and continue to increase above this threshold in the older population with time (Fig. 4a, b), signifying that transmission will continue in the community despite meeting the current TAS criteria focussed on monitoring infection in children only. Using the 95% EP threshold, on the other hand, will ensure that predicted recrudescence rates will be insignificant and infection will decline to zero over time for both the child and older populations once these thresholds are breached and MDA is discontinued.

Our conclusions are clearly dependent on the capacity of our data-driven modeling approach to capture real-word observations reasonably well. We carried out two sets of validation exercises to ascertain the validity of our predictions. The first assessed the predictive ability of our data-fitted local models for both reproducing baseline LF infection and for predicting the impacts of the annual MDAs applied in each of the present study sites on infection prevalence. The results shown in Fig. 1 and Supplementary Tables 4 and 5 highlight that apart from two villages (Piapung and Kirare sites), the estimated locally applicable LF models are able to reproduce both infection prevalence at baseline and the corresponding observed changes in the data due to the applied MDA interventions in our study villages, heightening the confidence that our data-driven modeling approach may provide a sound basis for the results presented here on the likely outcomes of using the WHO TAS criteria as a means for quantifying site-specific interruption of LF transmission. This predictive ability of our modeling approach to mimic real-world LF transmission and control dynamics is further underscored by the modeling results shown in Figs. 6 and 7 and in Supplementary Table 6 pertaining to field studies on the impact of supplementary VC^18^ and use of the WHO-proposed pre-TAS 1% mf threshold as a decision point for moving into the TAS phase^13^. Although carried out over relatively short intervention periods, the observations from these studies match model predictions indicating both that using a globally-fixed 1% mf prevalence may be insufficient to break LF transmission (Fig. 6) and that implementing VC into MDA programs can preserve the effects of MDA (Supplementary Table 6 and Fig. 7). These validations of the predictive performance of our data-driven modeling approach enhances the reliability of the results presented here, although analysis of longer-term data from more settings would further increase the validity of the conclusions regarding elimination thresholds, the TAS protocol, and interventions to ensure LF elimination made above. We note, again, that the elimination thresholds we have found could be substantially lower than those characterized in corresponding studies conducted using stochastic individual-based models. Arguably, this difference could be because the stochasticity defined at low-prevalence in such models may not be well characterized, which could result in overestimations of fade-outs^38^.

In summary, our study has shown on the one hand that using the right thresholds will be important for deciding if we are obtaining the right results, viz. in the present case whether applied LF interventions are able to sustainably interrupt community transmission of the parasite. On the other hand, we also show that assessing whether transmission elimination has occurred requires a deeper understanding of extinction dynamics following the meeting of these thresholds and how such dynamics interact with current or proposed LF interventions. Indeed, a key contribution of this work in this regard is to demonstrate that transitions or paths to LF elimination following the crossing of thresholds may be a slow process with a protacted period of transient dynamics involved during this regime shift. Together with the possibility that elimination thresholds may be significantly lower-valued, such a slow and drawn out transition to system extinction implies that detecting and affecting LF elimination will be challenging in the field. Although we show that including VC especially post-stoppage of MDA could provide a means to overcome these challenges^9,10^ if the WHO threshold is to be used, a problem is that this strategy will require both the implementation of VC into LF programs long-term once the 1% mf threshold is breached by MDA, and an assessment period for determining transmission cessation that is extended to at least between 10 and 15 years post-stoppage of MDA. This is a challenging task and questions LF control policies based on meeting the uncertain thresholds of a slow system with extended transition regimes. Setting of distinct targets may be alluring in policy-making but can have escalating costs if they are uncertain and exhibit complex behavior as shown in this paper. This implies that adding a pluralistic, integrated, approach during the endgame^15^, such as a strategy that reduces infection to low levels (say to 1% mf prevalence) before switching to long-term surveillance, mop-up treatments of detected residual infections diagnosed through existing disease monitoring programs, and the inclusion of VC, each enacted via a strengthened general heath system might be a more effective way for achieving LF elimination compared to the threshold-focused approach as practiced currently.

## Methods

### Data

Data on longitudinal changes in LF mf infection prevalences, and on MDA interventions carried out in six representative low, medium and high transmission sites (DokanTofa, Nigeria; Piapung, Nigeria; Mossasso, Mali; Kirare, Tanzania; Peneng, Papua New Guinea (PNG); Dozanso, Mali), were assembled from the published literature^39–43^ for use in calibrating and running simulations of the EPIFIL model in this study. The six sites were selected on the basis of providing data on the level of baseline mf prevalence, where DokanTofa and Piapung represent low-prevalence sites (mf prevalence 1–15%), Mossasso and Kirare embody medium-prevalence sites (mf prevalence 16–35%), and Peneng and Dozanso, high-prevalence sites (mf prevalence >35%), respectively, while also giving details on the required inputs for identifying the local LF models applicable to each individual site. These data inputs comprise information on the annual biting rate (ABR) and dominant mosquito genus, as well as MDA intervention details, including the relevant drug regimen used, frequency, and population coverages of the applied MDAs (Supplementary Table 2).

### The model

The EPIFIL LF transmission model used in this study has been previously described in full elsewhere^9,10,16^. The model simulates LF transmission in a population by accounting for key biological and intervention processes, such as impacts of vector density, the life cycle of the parasite, age-dependent exposure, density-dependent mechanisms, infection aggregation, and the outcomes arising from drug treatments as well as vector control. In brief, the population dynamics of filarial infection are represented as coupled differential equations describing changes in five state variables pertaining to infection in both human and vector host populations. In the human host, the pre-patent and patent worm loads (P and W, respectively), mf (M) and CFA (A) intensity and prevalence, and a measure of acquired immunity (I) to the parasite moderated by the total worm load (P+W=W_T_) are formulated as partial differential equations over time (t) and host age (a). In the mosquito host, the burden of infective L3 stage larvae (L) is represented by a single state variable (L*) formulated as an ordinary differential equation integrated over time (t) given that this stage is assumed to reach equilibrium quickly owing to the faster time scale of infection dynamics in the vector compared to the human. The model is vector genus-specific^9,28^ but given that the dominant vector genus in all the present study settings was *Anopheles* (Supplementary Table 2), the sub-model describing the uptake and development of mf into L3 larvae for these mosquitoes was applied in the present analysis. The structure, parameters, and functions of this anopheline-based EPIFIL model are detailed fully in Supplementary Methods and Supplementary Table 1 in Supplementary Information.

### Numerical stability analysis for quantifying infection thresholds and vector biting thresholds

A numerical stability analysis procedure, based on varying initial values of endemic infective larval density (L*) of each of the SIR-selected model parameter sets or vectors, was used to calculate the distribution of mf prevalence threshold s and the corresponding threshold biting rates (TBR) expected in each of study communities^9,10,16,28,30^. Briefly, in this method, we begin by progressively decreasing the vector-to-human density ratio, V/H, from its original value to a threshold value below which the model always converges to zero mf prevalence, regardless of the values of the endemic infective larval density L*. The product of the number of bites per mosquito, λ, and the newly found V/H value is termed as the threshold biting rate (TBR). Once the threshold biting rate is discovered, the model at TBR will settle to either a zero (trivial attractor) or non-zero mf prevalence depending on the starting value of L*. Thus, in the next step, while keeping all the model parameters unchanged, including the new V/H and by starting with a very low value of L* and progressively increasing it in very small step sizes we estimate the minimum L** below which the model predicts zero mf prevalence and above which the system progresses to a positive endemic infection state. Here, L** thus represents the L3 threshold density in the vector population^9^, whereas the corresponding mf and L3 prevalence at the L** value denotes the worm/mf or L3 prevalence threshold^9,23,28^. Note that this procedure can also be used to estimate the mf/L3 prevalence thresholds at the undisturbed ABR values in a site. In this case, the estimated threshold prevalences will always be lower than the corresponding maximal values at TBR^9,10^. The collections of mf or L3 threshold prevalences from the SIR-selected parameters in a site are then used to derive the infection extinction thresholds signifying various probabilities of elimination^29,30^. Note that in this study, we focused on the 95% elimination probability (EP) threshold value to serve as model-derived elimination targets for carrying out the intervention simulations described below.

### Calculating extinction and recrudescence probabilities

We calculated the probability that LF extinction has been achieved in each study site due to the applied intervention by quantifying the proportion of the best-fit SIR model prevalences that were declining or declined to zero (ie. models giving rise to mf prevalence curves with significant negative slopes) by the end of the 5 or 20-year simulation period following crossing of either the pre-TAS threshold of 1% mf or the predicted site-specific 95% EP threshold values, respectively. The recrudescence probability for a given study site was similarly calculated as the proportion of the total SIR-selected model runs that managed to revive and generate positive increases in mf prevalence (i.e., give rise to mf curves with significant positive slopes) by the end of each of the above simulation periods once MDAs are stopped. Finally, the curves having non-significant and fluctuating positive or negative slopes are considered as those exhibiting transient behavior.

### Modeling the impact of MDA and vector control interventions

Interventions were modeled by using the SIR-selected parameter vectors/models for simulating the impacts of both currently used as well as proposed MDA-based intervention strategies in reducing the observed baseline LF prevalence in each site to below either the global TAS (1% mf prevalence) or site-specific 95% EP thresholds. When simulating these interventions, the observed MDA regimens and coverages followed in each site were used (Supplementary Table 2), while MDA was assumed to target all residents aged 5 years and above. While the drug-induced mf kill rate and the duration of adult worm sterilization were fixed among the models (Supplementary Table 1), the worm kill rate was left as a free parameter to be estimated from the post-intervention data to account for uncertainty in this drug efficacy parameter. For making mf prevalence forecasts beyond the observations made in each site, predictions arising from the impacts of MDA simulated with and without vector control were carried out for 5 years and 20 years after the stoppage of MDA in each site. Three different MDA regimens: (i) annual MDA with ivermectin and albendazole (IVM+ALB) or diethylcarbamazine and ivermectin (IVM+DEC) as applied in each site, (ii) biannual MDA with the above regimens, and (iii) annual triple drug MDA (IDA: combined ivermectin, diethylcarbamazine and albendazole) were modeled with and without vector control in this study to provide a comparison of the effectiveness of these drug regimens for affecting LF elimination. In these simulations, MDAs are stopped after achieving either the 1% mf TAS threshold or the model-predicted 95% EP threshold in each modeled site but simulations of subsequent changes in mf prevalence with or without VC for the next 20 years were continued to evaluate the probability of LF elimination and the risk of recrudescence of the infection respectively over both the shorter 5-year TAS period and over the longer-term 20 years period (using the assessment methods for calculating the occurrence of either of these events described above). VC is modeled in terms of the impact of long-lasting insecticidal nets (LLINs) with 65% coverage following the equation given in ref. ^9^. The specific details concerning the different MDA-based scenarios investigated are as listed in Tables 1 and 2.

Note that the estimated 95% EP mf prevalence threshold at ABR was used when carrying out simulations of durations or timelines to break transmission by MDA alone strategies while the corresponding and comparatively higher 95% EP thresholds (obtaining at TBR) for this indicator was employed when modeling the impact of including VC into MDA programs^9^. The MDA plus MDA and vector control model formulations, parameters and functions used to carry out these simulations are as described previously and provided in Supplementary Information.

### Statistics and reproducibility

We used a data-model assimilation technique based on the Bayesian Melding (BM) algorithm to calibrate and identify locality-specific LF transmission models based on the baseline mf prevalence and vector biting intensity data observed in each of our study sites^9^. This is done by “melding” the observed baseline data from each site (Supplementary Table 2) with model-generated outputs in order to learn or parameterize models for describing the localized parasite transmission dynamics. The fitted models from each site were then used to quantify the various quantities of interest to this study, viz. estimations of mf thresholds and threshold biting rates, predictions of the impact of various MDA interventions with and without vector control on mf prevalence, and calculations of the probabilities of transmission interruption and recrudescence from using the WHO-set TAS thresholds versus model-derived threshold values once mf prevalences are forecast to cross below these thresholds in each study site.

The BM procedure begins by first specifying a range of plausible parameter values to generate distributions of parameter priors. We then randomly sample from those prior distributions to generate 200,000 parameter vectors, which are then used with the observed ABR in a site to generate predictions of baseline age-specific prevalences. The Sampling Importance Resampling (SIR) algorithm is then used to select *N* (typically *N* = 500) parameter vectors, *θ*, or models applicable to a site based on their likelihoods for describing the observed local baseline prevalence data. This BM fitting procedure normally relies on observed baseline age profiles of mf prevalence^9^, but, in the present analysis, these data were available only for DoakanTofa and Piapung, while overall community-level mf prevalences were available for the other sites (Mossasso, Kirare, Peneng, Dozanso). In this scenario, the observed overall prevalences from these sites were transformed into theoretical age-infection profiles using: (1) the national demographic profile applicable to the site in question, and (2) by conversion of the community-level mf prevalence to reflect either a plateau, concave or linear age-infection profile known typically to occur in LF endemic regions^15^. The derived age-prevalence infection data were then used in the model fitting procedures described above, which also effectively allowed the integration of partially observed data into the present LF model.

### Reporting summary

Further information on research design is available in the Nature Portfolio Reporting Summary linked to this article.

## Supplementary information

Peer Review File

Supplementary Information

Reporting Summary

## Data Availability

All the data used in this work are provided in the main text in different tables with proper citations.

## Code availability

All the MATLAB codes used in this work are available in GitHub: https://github.com/sharmas22/LF-TAS-project.git.
