# Supplementary material for: Evaluating elimination thresholds and stopping criteria for interventions against the vector-borne macroparasitic disease, lymphatic filariasis, using mathematical modelling
Source: Commun Biol. 2023 Feb 27;6:225. doi: 10.1038/s42003-022-04391-9 (PMC9971242; doi:10.1038/s42003-022-04391-9)
Supplement: Supplementary file 2 — Supplementary Information [file 42003_2022_4391_MOESM2_ESM.pdf]

Supplementary Information. EPIFIL model description, data used in model estimation, Monte Carlo p-values for pre- and post-MDA data, impact of interventions using either the 1% or 95% EP mf thresholds in the study sites, and details of the Sunish et al study.

Supplementary Methods

The mathematical model of LF transmission dynamics

We employed a genus specific mosquito-vectored transmission model of LF to carry out the modelling work in this study [1-7]. Briefly, the state variables of this hybrid coupled partial differential and differential equation model vary over age (a) and/or time (t), representing changes in the pre-patent worm burden per human host $(P(a,t))$, adult worm burden per human host $(W(a,t))$, the microfilariae (mf) level in the human host modified to reflect infection detection in a 1 mL blood sample $(M(a,t))$, the average number of infective L3 larval stages per mosquito (L), and a measure of immunity $(I(a,t))$ developed by human hosts against L3 larvae; and intensity of Circulating Filarial Antigen (CFA) (denoted by $A(a, t)$). The state equations comprising this model are:

$$\frac{\partial P}{\partial t}+\frac{\partial P}{\partial a}=\lambda\frac{V}{H}h(a)\Omega(a,t)-\mu P(a,t)-\lambda\frac{V}{H}h(a)\Omega(a,t-\tau)\varsigma$$
$$\frac{\partial W}{\partial t}+\frac{\partial W}{\partial a}=\lambda\frac{V}{H}h(a)\Omega(a,t-\tau)\varsigma-\mu W(a,t)$$
$$\frac{\partial M}{\partial t}+\frac{\partial M}{\partial a}=\alpha s\phi[W(a,t),k]W(a,t)-\gamma M(a,t)$$
$$\frac{\partial I}{\partial t}+\frac{\partial I}{\partial a}=W_T(a,t)-\delta I(a,t)$$
$$\frac{\partial A}{\partial t}+\frac{\partial A}{\partial a}=\alpha_2 W(a,t)-\gamma_2 A(a,t)$$
$$\frac{dL}{dt}=\lambda\kappa g\int\pi(a)\big(1-f[M(a,t)]\big)da-\sigma L-\lambda\psi_1 L$$
$$L^*=\frac{\lambda\kappa g\int\pi(a)\big(1-f[M(a,t)]\big)da}{\sigma+\lambda\psi_1}$$

The above equations involve partial derivatives of four state variables (P - pre-patent worm load; W - adult worm load; M - microfilaria intensity; I - immunity to acquiring new infection due to the pre-existing total worm load; A - intensity of CFA where $W_T = W(a,t) + P(a,t)$). Given the faster time scale of infection dynamics

in the vector compared to the human host, the infective L3-stage larval density in mosquito population is modelled by an ordinary differential equation essentially reflecting the significantly faster time-scale of the infection dynamics in the vector hosts. This allows us to make the simplifying assumption that the density of infective stage larvae in the vector population reaches a dynamic equilibrium (denoted by L^*) rapidly[1, 2, 5, 8, 9]. This basic coupled immigration-death structure of the model as well as its recent extensions has been extensively discussed previously[1-3, 5, 8, 9]. The effects of worm patency are captured by considering that at any time *t*, human individuals of age less than or equal to the pre-patency period, τ, will have no adult worms or mf, and the rate at which pre-patent worms survive to become adult worms in these individuals at $a > \tau$ is given by $\zeta = \exp(-\mu\tau)$. The term enables us to account for the different establishment and development rates of the incoming L3-stage larvae as adult worms depending on the genus of mosquito vectors as expressed below:

$$f[M(a,t)] = \left[\frac{2}{\left[1+\frac{M(a,t)}{k}\left(1-exp\left[-\frac{r}{\kappa}\right]\right)\right]^{k}} - \frac{1}{\left[1+\frac{M(a,t)}{k}\left(1-exp\left[-\frac{2r}{\kappa}\right]\right)\right]^{k}}\right]$$ for mosquitoes of *Anopheline* genus;

$$f[M(a,t)] = \left(1+\frac{M(a,t)}{k}\left(1-exp\left[-\frac{r}{\kappa}\right]\right)\right)^{-k}$$ for mosquitoes of *Culicine* genus.

In the above, $k[= k_0 + k_{Lin}M]$ is the shape parameter of the negative binomial distribution on the mf uptake whereas r and κ respectively the rate of initial increase and the maximum level of L3 larvae. See Supplementary Table 1 for the description of all the model parameters and functions.

Supplementary Table 1: **Description of EPIFIL model parameters and functions.**

Parameter	Definition (*units*)	Range	Refs
λ	Number of bites per mosquito (*per month*)	[5, 15]	1, 2, 5, 10, 11
τ	Pre-patency period	[1, 9]	12
s	Proportion of female worms	0.5	-
μ	The worm mortality rate (*per month*)	[0.008, 0.018]	1, 2, 5, 13-16
α	Production rate of microfilariae per worm (*per month*)	[0.25, 1.5]	1, 2, 5, 17
γ	The death rate of the microfilariae (*per month*)	[0.08, 0.12]	1, 5, 15, 17
α_2	Production rate of CFA (*per worm per month*)	[2, 8]	This study
γ_2	Decay rate of CFA (*per month*)	[0.4, 0.5]	This study
g	Proportion of mosquitoes which pick up infection when biting an infected host	[0.251, 0.485]	1, 5, 18
κ	Maximum level of L3 given Mf density	[3, 5]	1, 5
k_0	The basic location parameter of negative binomial distribution used in aggregation parameter ($k = k_0 + k_{Lin} M$)	[0.000036, 0.000775]	1, 5, 19, 20
δ	Immunity waning rate (*per month*)	[0.001, 0.01]	1, 5
V/H	Ratio of number of vector to hosts	$MBR^{\#} / \lambda$	data
k_{Lin}	The linear rate of increase in the aggregation parameter defined above	[0.00000024, 0.282]	1, 5, 19, 20
σ	Death rate of mosquitoes (*per month*)	[1.5, 8.5]	1, 5, 20
ψ_1	Proportion of L3 leaving mosquito per bite	[0.1, 0.8]	17
ψ_2	The establishment rate[1]	[0.00003, 0.00364]	1, 2, 5, 21
H_{Lin}	A threshold value used in *h(a)* to adjust the rate at which individuals of age *a* are bitten: linear rise from 0 at age zero to 1 at age H_{Lin} in years. $h(a) = a / H_{Lin}$ for $a < H_{Lin}$; $h(a) = 1$ for $a \geq H_{Lin}$	[240, 360] months	1, 5, 9
r	Gradient of Mf uptake[2]	[0.04, 0.25]	1, 5
c	Strength of acquired immunity	[0.015, 0.025]	1, 5
I_C	Strength of immunosuppression[3]	[0.5, 5.5]	1, 5
S_C	Slope of immunosuppression function[4] (*per worm/month*)	[0.01, 0.20]	1, 5
	Intervention-related parameters		
ω	Worm killing efficacy of drug (instantaneous)	dependent on drug regimen	3
ε	Microfilariae killing efficacy of drug (instantaneous)	dependent on drug regimen	3
δ_{reduc}	Reduction in the worm's fecundity over a period of time *p* due to drug	dependent on drug regimen	3
p	A time period during which the drug remains efficacious in reducing the fecundity of the surviving adult worms	dependent on drug regimen	3
C	Percentage of the population administered the drug	data	data
MBR_{VC}	Vector control (VC) modifies V/H ($= MBR/\lambda$) where $MBR_{VC} = MBR_0 \exp[a_1 t]$, with $a_1 < 0$ for $\forall t$ when VC is implemented, otherwise $a_1 > 0$.	data and estimates	19, 20
**Description**	**Mathematical expressions of the functions**	**Parameters**	
Probability that an individual is of age *a* $\pi(a)$	$\pi(a) = A_0 \exp[-B_0 a]$	Human age *a* in month, A_0 and B_0 estimated from country demographic data	1, 5, 9

Larvae establishment rate (modified by acquired immunity) ***Ω(a,t)***	$L^{*}\psi_1\psi_2 g_1(I) g_2(W_T)$	ψ_1 - proportion of L3 leaving mosquito per bite; ψ_2 - the establishment rate[1]	-
Adult worm mating probability ***ϕ(W,k)***	$1-\left(1+\frac{W}{2k}\right)^{-(1+k)}$	*k* – negative binomial aggregation parameter	2, 5, 22
Immunity to larval establishment ***g₁(I)***	$\frac{1}{1+cI}$	*c* – strength of immunity to larval establishment	1, 5
Host immunosuppression ***g₂(W_T)***	$\frac{1+I_C S_C W_T}{1+S_C W_T}$	I_C – strength of immunosuppression; S_C – slope of immunosuppression	1, 5

[1]The proportion of L3-stage larvae infecting human hosts that survive to develop into adult worms[2].
[2]The gradient of Mf uptake *r* is a measure of the initial increase in the infective L3 larvae uptake by vector as *M* increases from 0[2, 9].
[3] The facilitated establishment rate of adult worms due to parasite-induced immunosuppression in a heavily infected human host
[4] The initial rate of increase by which the strength of immunosuppression is achieved as *W* increases from 0[23].
Note MBR (monthly biting rate) serves as an input to initialize the model, measured as mosquito bites per person per month, the value of which may be obtained from entomological surveys conducted in study sites. In the absence of the observed MBR value, the model has been adapted to estimate it from the community-level Mf prevalence data.

Modeling intervention by mass drug administration

Intervention by mass drug administration was modeled based on the assumptions that anti-filarial treatment with a combination drug regimen act by killing certain fractions of the populations of adult worms and microfilariae instantly after the drug administration[24]. These effects are incorporated into the basic model by calculating the population sizes of worms and microfilariae as follows:

$$\left.\begin{aligned} P(a,t+dt) &= (1-\omega C)P(a,t) \\ W(a,t+dt) &= (1-\omega C)W(a,t) \\ M(a,t+dt) &= (1-\varepsilon C)M(a,t) \end{aligned}\right\} \quad \text{at time } t = T_{MDA_i}$$

where *dt* is a short time period since the *i*th MDA was administered. During this short time interval, a given proportion of adult worms and microfilariae are instantly removed. The parameters ω and ε are drug killing efficacy rates for the two life stages of the parasite while the parameter *C* represents the MDA coverage. Apart from instantaneous killing of microfilariae, the drug continues to kill the newly reproduced mf by any surviving adult worms at a rate δ_{reduc} for a period of time, *p*. We model this effect as follows:

$$\frac{\partial M(a,t)}{\partial t}+\frac{\partial M(a,t)}{\partial a}=(1-\delta_{reduc}C)s\alpha\phi(W(a,t),k)W(a,t)-\gamma M(a,t)\,, \quad \text{for } T_{MDA_i}<t\le T_{MDA_i}+p$$

We simulated LF intervention by running the model with fixed values of ε, δ_{reduc}, and p (here $\delta_{reduc}=1$) for MDA coverage levels given by data. The worm-kill parameter, ω, was drawn from a uniform prior distribution such that the post-intervention data could inform this efficacy value. The first MDA round was implemented in the model by affecting the population sizes of worms and microfilariae from the baseline fits, and then the intervention is simulated forward in time for a number of years, with subsequent MDA rounds implemented annually.

Modeling intervention by Vector Control

In addition to MDA, we also modeled the added effect of long-lasting insecticidal nets (LLINs) as described previously[6]. The impact of LLINs with three main actions against mosquito biting was modelled: 1) deterrence from entering the home (efficacy η_1), 2) inhibition of their ability to feed on humans (efficacy η_2), and 3) killing them (efficacy η_3)[25,26]. To capture these effects, which decay over time as the larvicide efficacy declines exponentially at rate Λ, we adjust the term V/H to be appropriately modified according to the population coverage for LLINs (C_{LLIN}):

$$\frac{V}{H}(1-\eta_1\exp(-\Lambda t)C_{LLIN})(1-\eta_2\exp(-\Lambda t)C_{LLIN})(1-\eta_3\exp(-\Lambda t)C_{LLIN})$$

Supplementary Table 2: Baseline mf prevalence, ABR, and MDA data in the six LF endemic study sites.

Setting*	Site	Mosquito Genus	ABR[b]	Baseline mf prevalence	Regimen (Efficacy[c])	Average MDA coverage /Observed MDA rounds
Low	DokanTofa, Nigeria [27]	Anopheles	300-5000	5.0%	IVM+ALB (99/9)	76% /7
	Piapung, Nigeria [27]	Anopheles	300-5000	9.9%	IVM+ALB (99/9)	77% /7
Medium	Missasso, Mali [28]	Anopheles	605.9	20.2%	IVM+ALB (99/9)	75% /6
	Kirare, Tanzania [29]	Anopheles[a]	2090	26.1%	IVM+ALB (99/9)	66% /6
High	Peneng, PNG [5]	Anopheles	8194	66.7%	DEC+IVM (95/6)	69% /5
	Dozanso, Mali [28]	Anopheles	605.9	40.0%	IVM+ALB (99/9)	75% /6

*Low: mf prevalence 1%-15%, Medium: mf prevalence 16%-35%, High: mf prevalence >35%

[a]Transmission in Kirare is by both Anopheles and Culex mosquitoes, but models based on the dominant species (Anopheles) were used in this study. The ABR represents the combined biting rate.

[b]In the model simulations, the prior ABR range was informed by the observed ABRs reported here.

[c]Drug efficacy figures denote the proportionate instantaneous adult worm and mf kill rates and the duration of adult worm sterilization in months.

Supplementary Table 3: Monte Carlo p-values for pre- and post-MDA data.

	Age-stratified and overall Monte Carlo p-values for baseline data								
	**0-9**	**10-19**	**20-29**	**30-39**	**40-49**	**50-59**	**60-69**	**70-79**	**Overall**
DokanTofa	0.012	0.988	0.396	0.882	0.966	0.677	0.535	0.111	0.135
Piapung	0.026	0.055	0.031	0.315	0.315	0.002	0.48	0.125	0.011
Missasso									0.149
Kirare									0.022
Peneng									0.878
Dozanso									0.215
	MDA round wise and overall Monte Carlo p-values for post MDA data								
	**1st**	**2nd**	**3rd**	**4th**	**5th**	**6th**	**7th**	**8th**	**Overall**
DokanTofa			1.0	0.974	1.0	0.907	0.977		1.0
Piapung			1.0	1.0	1.0		1.0		1.0
Missasso	0.91						0.884		0.815
Kirare	0.99	0.994	1.0		1.0	0.986	0.998		0.972
Peneng	0.761	0.938	0.249	0.122	0.508	0.273			0.671
Dozanso	0.952						0.878		0.865

Monte Carlo p-values: MONTE CARLO significance test procedures consist of the comparison of the observed data with random samples generated in accordance with the hypothesis being tested. The p-value is the probability of obtaining results at least as extreme as the observed results of a statistical hypothesis test, assuming that the null hypothesis is correct. A smaller p-value means that there is stronger evidence in favour of the alternative hypothesis. $p > 0.05$ is the probability that the null hypothesis is true. A statistically significant test result ($p \leq 0.05$) means that the test hypothesis is false or should be rejected.

Supplementary Table 4: Observed and model predicted post-MDA outcomes based on using the WHO recommended 1% mf threshold for the six LF infected sites.

Settings	Site	No. of annual MDA rounds observed	mf prevalence following interventions	Mean No. of years to reach 1% mf (model predicted)	Probability of elimination (%) 5years after crossing 1% mf threshold	Probability of recrudescence (%) 5years after crossing 1% mf threshold
Low	DokanTofa, Nigeria	7	0.4%	3	1	21
	Piapung, Nigeria	7	2.1%	4	0	15
Medium	Missasso, Mali	6	0.0%	5	1	34
	Kirare, Tanzania	6	2.7%	7	9	55
High	Peneng, PNG	5	3.7%	9	41	35
	Dozanso, Mali	6	0.0%	6	12	34

Supplementary Table 5: Model predicted post-MDA outcomes for model predicted 95% EP mf thresholds for the six LF infected sites.

Settings	Site	95% mf EP threshold values for ABR (for TBR)	k_0~	k_{lin}~	Mean no. of years to reach 95% mf EP threshold (model predicted)	Probability of elimination (%) 5years after crossing model predicted 95% EP threshold	Probability of recrudescence (%) 5years after crossing model predicted 95% mf EP threshold
Low	DokanTofa, Nigeria	0.003109 (0.031017)	0.00005-0.0011	0.0025-0.3455	11	64	1
	Piapung, Nigeria	0.001550 (0.041388)	0.00004-0.0011	0.0109-0.3835	13	31	0
Medium	Missasso, Mali	0.007500 (0.021637)	0.00006-0.0012	0.0074-0.157	15	72	0
	Kirare, Tanzania	0.001799 (0.037083)	0.00006-0.0011	0.0049-0.3219	18	73	1
High	Peneng, PNG	0.004534 (0.065918)	0.00006-0.00076	0.0154-0.2508	19	98	1
	Dozanso, Mali	0.001101 (0.018659)	0.00011-0.0011	0.0042-0.3142	16	54	0

~ Parameter values for the linear infection aggregation function estimated in each study site. Note that these low values indicate a high level of infection clustering in each community, which constitute a major reason for the corresponding low valued 95% EP thresholds estimated for each site.

Supplementary Table 6: Pre- and post-treatment mf prevalence data for two groups of villages in Tirukoilur, India.

**Treatment Groups**	**M&E data [30]**		
	**Pre-treatment (1994 October-December) mf positive (%) (no. of sample)**	**First survey (1997 October-December) mf positive (%) (no. of sample)**	**Second survey (1999 April-June) mf positive (%) (no. of sample)**
Group A (MDA alone for 1995 (June-August) and 1996 (July-September)	15.19 (724)	1.81 (609)	4.74 (591)
Group B (MDA+VC for 1995 (June-August) and 1996 (July-September) and VC up to 1998 (December))	15.09 (795)	1.24 (645)	2.08 (673)

Supplementary Figure 1: Impact of annual MDA, biannual MDA and annual IDA on L3 prevalence in Piapung for the model predicted 95% EP mf threshold. Red curves indicate the model predicted L3 curves not having significant positive or negative slopes (considered as transient curves) whereas gray lines indicate the model predictions having significant positive or negative slopes. Numbers in red color indicate the percentage of transient curves. Blue, black, and green dotted vertical lines indicate the times to cross the 95% EP L3 threshold, 95% EP mf threshold, and 5years after reaching 95% EP threshold respectively, whereas the black and blue dotted horizontal lines are indicating 95% EP mf and L3 thresholds respectively.

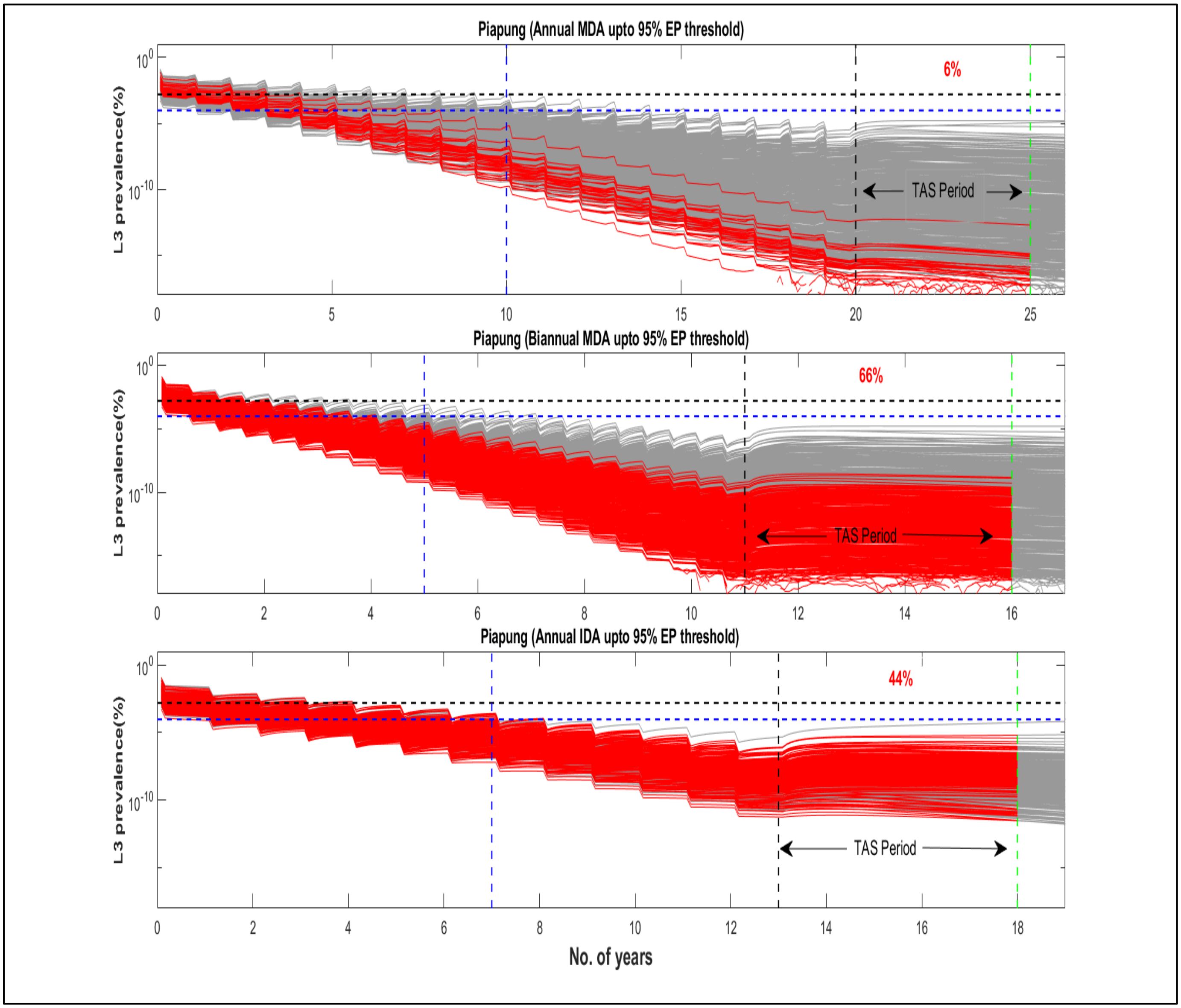

Supplementary References

1. Gambhir, M. & Michael, E. Complex ecological dynamics and eradicability of the vector borne macroparasitic disease, lymphatic filariasis. *PLoS One* **3**, e2874 (2008).

2. Gambhir, M. Geographic and ecologic heterogeneity in elimination thresholds for the major vector-borne helminthic disease, lymphatic filariasis. *BMC Biol.* **8**, 1 (2010).

3. Michael, E. et al. Mathematical modelling and the control of lymphatic filariasis. *Lancet Infect. Dis.* **4**, 223-234 (2004).

4. Michael, E., Malecela-Lazaro, M. N., Kabali, C., Snow, L. C. & Kazura, J. W. Mathematical models and lymphatic filariasis control: endpoints and optimal interventions. *Trends Parasitol.* **22**, 226-233 (2006).

5. Singh, B. K. et al. Sequential modelling of the effects of mass drug treatments on anopheline mediated lymphatic filariasis infection in Papua New Guinea. *PLoS One* **8**, e67004 (2013).

6. Singh, B. K. & Michael, E. Bayesian calibration of simulation models for supporting management of the elimination of the macroparasitic disease, Lymphatic Filariasis. *Parasit Vectors* **8**, 1-26 (2015).

7. Michael, E. & Singh, B. K. Heterogeneous dynamics, robustness/fragility trade-offs, and the eradication of the macroparasitic disease, lymphatic filariasis. *BMC Med.* **14**, 1 (2016).

8. Chan, M. S. et al. Epifil: a dynamic model of infection and disease in lymphatic filariasis. *Am. J. Trop. Med. Hyg.* **59**, 606-614 (1998).

9. Norman, R. et al. EPIFIL: the development of an age-structured model for describing the transmission dynamics and control of lymphatic filariasis. *Epidemiol. Infect.* **124**, 529-541 (2000).

10. Rajagopalan, P. Population dynamics of culex pipiens fatigans, the filariasis vector, in pondicherry: influence of climate and environment. *Proc. Ind. Nat. Science Acad. B* **46**, 745-752 (1980).

11. Subramanian, S., Manoharan, A., Ramaiah, K. D. & Das, P. K. Rates of acquisition and loss of Wuchereria bancrofti infection in Culex quinquefasciatus. *Am. J. Trop. Med. Hyg.* **51**, 244-249 (1994).

12. Scott, A. L. & Nutman, T. Lymphatic-dwelling filariae. Lymphatic filariasis., 5-39 (2000).

13. Vanamail, P., Subramanian, S., Das, P. K., Pani, S. P. & Rajagopalan, P. K. Estimation of fecundic life span of Wuchereria bancrofti from longitudinal study of human infection in an endemic area of Pondicherry (south India). *Indian J. Med. Res.* **91**, 293-297 (1990).

14. Evans, D. B., Gelband, H. & Vlassoff, C. Social and economic factors and the control of lymphatic filariasis: a review. *Acta Trop.* **53**, 1-26 (1993).

15. Ottesen, E. & Ramachandran, C. Lymphatic filariasis infection and disease: control strategies. *Parasitol. Today* **11**, 129-130 (1995).

16. Vanamail, P. et al. Estimation of the fecund life span of Wuchereria bancrofti in an endemic area. *Trans. R. Soc. Trop. Med. Hyg.* **90**, 119-121 (1996).

17. Hairston, N. G. & de Meillon, B. On the inefficiency of transmission of Wuchereria bancrofti from mosquito to human host. Bull. *World Health Organ.* **38**, 935-941 (1968).

18. Subramanian, S. et al. The relationship between microfilarial load in the human host and uptake and development of Wuchereria bancrofti microfilariae by Culex quinquefasciatus: a study under natural conditions. *Parasitology* **116**, 243-255 (1998).

19. Subramanian, S., Pani, S., Das, P. & Rajagopalan, P. Bancroftian filariasis in Pondicherry, south India: 2. Epidemiological evaluation of the effect of vector control. *Epidemiol. Infect.* **103**, 693-702 (1989).

20. Das, P. et al. Bancroftian filariasis in Pondicherry, south India–epidemiological impact of recovery of the vector population. *Epidemiol. Infect.* **108**, 483-493 (1992).

21. Ho, B. C. & Ewert, A. Experimental transmission of filarial larvae in relation to feeding behaviour of the mosquito vectors. *Trans. R. Soc. Trop. Med. Hyg.* **61**, 663-666 (1967).

22. May, R. M. Togetherness among schistosomes: its effects on the dynamics of the infection. *Math. Biosci.* **35**, 301-343 (1977).

23. Duerr, H., Dietz, K. & Eichner, M. Determinants of the eradicability of filarial infections: a conceptual approach. *Trends Parasitol*. **21**, 88-96 (2005).

24. Michael, E. et al., Quantifying the value of surveillance data for improving model predictions of lymphatic filariasis elimination. *PLoS Negl. Trop. Dis.***12(10)**, e0006674 (2018).

25. Griffin, J. T. et al. Reducing Plasmodium falciparum malaria transmission in Africa: a model-based evaluation of intervention strategies. *PLoS Med.* **7**, e1000324 (2010).

26. Okumu, F. O. & Moore, S. J. Combining indoor residual spraying and insecticide-treated nets for malaria control in Africa: a review of possible outcomes and an outline of suggestions for the future. *Malar. J.* **10**, 1 (2011).

27. Richards, F. O. et al. Epidemiological and entomological evaluations after six years or more of mass drug administration for lymphatic filariasis elimination in Nigeria. *PLoS Negl. Trop. Dis.* **5**, e1346 (2011).

28. Coulibaly, Y. I. *et al.* The Impact of Six Annual Rounds of Mass Drug Administration on Wuchereria bancrofti Infections in Humans and in Mosquitoes in Mali. *Am. J. Trop. Med. Hyg.* **93(2)**, 356–360 (2015).

29. Simonsen, P. E. et al. Lymphatic filariasis control in Tanzania: effect of repeated mass drug administration with ivermectin and albendazole on infection and transmission. *PLoS Negl. Trop. Dis.* **4**, e696 (2010).

30. Sunish, I. *et al.* Resurgence in filarial transmission after withdrawal of mass drug administration and the relationship between antigenaemia and microfilaraemia–a longitudinal study. *Trop. Med. Int. Health* **7**, 59-69 (2002).
