# Supplementary material for: Evaluating elimination thresholds and stopping criteria for interventions against the vector-borne macroparasitic disease, lymphatic filariasis, using mathematical modelling
Source: Commun Biol. 2023 Feb 27;6:225. doi: 10.1038/s42003-022-04391-9 (PMC9971242; doi:10.1038/s42003-022-04391-9)
Supplement: Supplementary file 3 — Reporting Summary [file 42003_2022_4391_MOESM3_ESM.pdf]

nature portfolio

Corresponding author(s): Dr. Swarnali Sharma
Last updated by author(s): Nov 30, 2022

Reporting Summary

Nature Portfolio wishes to improve the reproducibility of the work that we publish. This form provides structure for consistency and transparency in reporting. For further information on Nature Portfolio policies, see our Editorial Policies and the Editorial Policy Checklist.

Statistics

For all statistical analyses, confirm that the following items are present in the figure legend, table legend, main text, or Methods section.

n/a	Confirmed	
☐	☒	The exact sample size (*n*) for each experimental group/condition, given as a discrete number and unit of measurement
☒	☐	A statement on whether measurements were taken from distinct samples or whether the same sample was measured repeatedly
☒	☐	The statistical test(s) used AND whether they are one- or two-sided<br>*Only common tests should be described solely by name; describe more complex techniques in the Methods section.*
☒	☐	A description of all covariates tested
☒	☐	A description of any assumptions or corrections, such as tests of normality and adjustment for multiple comparisons
☐	☒	A full description of the statistical parameters including central tendency (e.g. means) or other basic estimates (e.g. regression coefficient) AND variation (e.g. standard deviation) or associated estimates of uncertainty (e.g. confidence intervals)
☐	☒	For null hypothesis testing, the test statistic (e.g. *F*, *t*, *r*) with confidence intervals, effect sizes, degrees of freedom and *P* value noted<br>*Give P values as exact values whenever suitable.*
☐	☒	For Bayesian analysis, information on the choice of priors and Markov chain Monte Carlo settings
☒	☐	For hierarchical and complex designs, identification of the appropriate level for tests and full reporting of outcomes
☒	☐	Estimates of effect sizes (e.g. Cohen's *d*, Pearson's *r*), indicating how they were calculated

Our web collection on statistics for biologists contains articles on many of the points above.

Software and code

Policy information about availability of computer code

Data collection	We have collected data from the published literatures and provided in the main text of the paper in the form of different tables with proper citations.
Data analysis	We have modelled the data using MATLAB code provided in https://github.com/sharmas22/LF-TAS-project.git

For manuscripts utilizing custom algorithms or software that are central to the research but not yet described in published literature, software must be made available to editors and reviewers. We strongly encourage code deposition in a community repository (e.g. GitHub). See the Nature Portfolio guidelines for submitting code & software for further information.

Data

Policy information about availability of data

All manuscripts must include a data availability statement. This statement should provide the following information, where applicable:

- Accession codes, unique identifiers, or web links for publicly available datasets
- A description of any restrictions on data availability
- For clinical datasets or third party data, please ensure that the statement adheres to our policy

All the data used in this work are provided in the main text in different tables with proper citation.

Human research participants

Policy information about studies involving human research participants and Sex and Gender in Research.

Reporting on sex and gender	NA
Population characteristics	NA
Recruitment	NA
Ethics oversight	NA

Note that full information on the approval of the study protocol must also be provided in the manuscript.

Field-specific reporting

Please select the one below that is the best fit for your research. If you are not sure, read the appropriate sections before making your selection.

☐ Life sciences ☐ Behavioural & social sciences ☒ Ecological, evolutionary & environmental sciences

For a reference copy of the document with all sections, see nature.com/documents/nr-reporting-summary-flat.pdf

Ecological, evolutionary & environmental sciences study design

All studies must disclose on these points even when the disclosure is negative.

Study description	We used a data-model assimilation technique based on the Bayesian Melding (BM) algorithm to calibrate and identify locality-specific LF transmission models based on the baseline mf prevalence data observed in each of our study sites: DokanTofa, Nigeria; Piapung, Nigeria; Mossasso, Mali; Kirare, Tanzania; Peneng, Papua New Guinea (PNG); Dozanso, Mali. The fitted models from each site were then used to quantify the various quantities of interest to this study, viz. estimations of mf thresholds and threshold biting rates, predictions of the impact of various MDA interventions with and without vector control on mf prevalence, and calculations of the probabilities of transmission interruption and recrudescence from using the WHO-set TAS thresholds versus model-derived threshold values once mf prevalences are forecast to cross below these thresholds in each study site.
Research sample	Secondary published data from two low mf Prevalence sites: DokanTofa, Nigeria; Piapung, Nigeria; Two medium mf prevalence sites: Mossasso, Mali; Kirare, Tanzania; two high mf prevalence sites: Peneng, Papua New Guinea (PNG); Dozanso, Mali, reflecting endemicity variation and providing all other relevant data for modelling, viz. MDA coverage/duration, ABR, and pre-post mf prevalences were used in the study.
Sampling strategy	The BM procedure begins by first specifying a range of plausible parameter values to generate distributions of parameter priors. We then randomly sample from those prior distributions to generate 200,000 parameter vectors, which are then used with the observed ABR in a site to generate predictions of baseline age-specific pevalences. The Sampling Importance Resampling (SIR) algorithm is then used to select N (typically N=500) parameter vectors, θ, or models applicable to a site based on their likelihoods for describing the observed local baseline prevalence data.
Data collection	We collected data from the published literature and provide them in the main text of the paper in the form of tables with the relevant citation.
Timing and spatial scale	Data was collected for communities of DokanTofa and Piapung in 2002, for Missasso in 2002, for Kirare in 2004, for Peneng in 1994 and for Dozanso in 2002.
Data exclusions	No Data was excluded.
Reproducibility	NA
Randomization	NA – as described above secondary published providing all relevant information for supporting the modelling work was used in this study.
Blinding	NA

Did the study involve field work? ☐ Yes ☒ No

Reporting for specific materials, systems and methods

We require information from authors about some types of materials, experimental systems and methods used in many studies. Here, indicate whether each material, system or method listed is relevant to your study. If you are not sure if a list item applies to your research, read the appropriate section before selecting a response.

Materials & experimental systems

n/a	Involved in the study
☒	☐ Antibodies
☒	☐ Eukaryotic cell lines
☒	☐ Palaeontology and archaeology
☒	☐ Animals and other organisms
☒	☐ Clinical data
☒	☐ Dual use research of concern

Methods

n/a	Involved in the study
☒	☐ ChIP-seq
☒	☐ Flow cytometry
☒	☐ MRI-based neuroimaging
